# Energy-efficient superparamagnetic Ising machine and its application to traveling salesman problems

**DOI:** 10.1038/s41467-024-47818-z

**Published:** 2024-04-24

**Authors:** Jia Si, Shuhan Yang, Yunuo Cen, Jiaer Chen, Yingna Huang, Zhaoyang Yao, Dong-Jun Kim, Kaiming Cai, Jerald Yoo, Xuanyao Fong, Hyunsoo Yang

**Affiliations:** 1 Department of Electrical and Computer Engineering, National University of Singapore, Singapore, Singapore; 2 Key Laboratory for the Physics and Chemistry of Nanodevices and Center for Carbon-based Electronics, School of Electronics, Peking University, Beijing, China

**Keywords:** Electrical and electronic engineering, Magnetic devices

## Abstract

The growth of artificial intelligence leads to a computational burden in solving non-deterministic polynomial-time (NP)-hard problems. The Ising computer, which aims to solve NP-hard problems faces challenges such as high power consumption and limited scalability. Here, we experimentally present an Ising annealing computer based on 80 superparamagnetic tunnel junctions (SMTJs) with all-to-all connections, which solves a 70-city traveling salesman problem (TSP, 4761-node Ising problem). By taking advantage of the intrinsic randomness of SMTJs, implementing global annealing scheme, and using efficient algorithm, our SMTJ-based Ising annealer outperforms other Ising schemes in terms of power consumption and energy efficiency. Additionally, our approach provides a promising way to solve complex problems with limited hardware resources. Moreover, we propose a cross-bar array architecture for scalable integration using conventional magnetic random-access memories. Our results demonstrate that the SMTJ-based Ising computer with high energy efficiency, speed, and scalability is a strong candidate for future unconventional computing schemes.

## Abstract (web summary)

Ising machines are promising for solving NP-hard problems, but current implementations have power consumption and scalability challenges. Si et al. implement an Ising machine consisting of 80 superparamagnetic tunnel junctions with all-to-all connections and apply it to a large-scale travelling salesman problem.

## Introduction

The demands for future data-intensive and energy-efficient computing tasks overwhelm the computational power of conventional von Neumann architectures^1^. For example, NP-hard problems are often encountered in combinatorial optimizations^2^, resource allocation^3^, cryptography^4^, finance^5^, image processing^6^, tour planning^7^, and job sequencing^8^, and their computational time and hardware resources increase exponentially with the problem size, which makes them very difficult or impossible to be solved by conventional computers in a finite time. These problems can be mapped to the Ising model, a mathematical model to characterize interactions between magnetic spins^9^. The dynamics of the model is algorithm- based, i.e. by constructing a proper coupling matrix and allowing the system to evolve utilizing an intrinsic convergence property of the Ising model, the ground state could be obtained as a solution to the corresponding problems. However, as the system might be trapped in many local minima, the annealing process has usually been adopted in Ising computers to address such limitations. It is commonly agreed that adding fluctuations prevents the Ising computer from being stuck at the local minima.

Efficient algorithms and hardware systems for finding an optimal or near-optimal solution of an Ising model at a fast speed and low power have been sought. Adiabatic quantum computing (AQC)^10,11^ and quantum computing^12–15^ based on superconducting qubits are capable of converging the Ising model by tunneling out of local minima to the global minima. A 100-node Maxcut problem was solved using a quantum computer of 2048 spins with huge power consumption^16^. Besides the high cost and complexity of cryogenic temperature, this proof-of-concept system was limited by the sparse connections only between the nearest neighbors, which leads to sub-optimal outcomes^17^. Simulated annealing based on CMOS implementations was exploited for parallel Ising computing, including central processing units (CPU)^18,19^, graphics processing units (GPU)^20^, and field-programmable gate array (FPGA)^21,22^. These hardware have reported as large as 16,384 spins, however, it requires huge hardware resources for generating random numbers to introduce stochasticity to escape from the local minima^4,18,23,24^. Coherent Ising machine (CIM) is an optical scheme with competitive energy efficiency. However, it requires a long fiber ring cavity and relies on external FPGA for implementing coupling^25,26^. The temporal multiplexing process is also time-consuming and hard to expand to large systems. Recently, experiments and simulation works have investigated various devices to emulate the behavior of Ising spins by taking advantage of their intrinsic physics. An 8-spin asynchronous probabilistic computer based on superparamagnetic tunnel junctions for solving integer factorization tasks of values up to 945 was demonstrated^4^. SPICE simulations of 16-city TSP using simulated annealing method were presented^27^. Other works such as 8-spin phase-transition nano-oscillators^28^, multiferroic oxide devices with a high thermal stability^29^, and magnetoresistive random access memory (MRAM)^30,31^ have also conceptually proved that spin-based devices are suitable for representing Ising units. However, these works have encountered challenges in either partially-connected Ising spins or small scalability which limit the Ising computer from solving practical problems.

TSP discussed in this paper is a well-known problem which is much beyond the limitation of locally connected Ising models. Other combinatorial optimization problems, such as knapsack problems, coloring problems, and number partitioning, need all-to-all connection to satisfy specific constraints^9^. In practice, an additional graph embedding process is often required when mapping to 2-dimensional CMOS circuitry which only considered the coupling between adjacent spins^32–34^. Since the embedding increases the required number of auxiliary spins and causes spin connections to change, the annealing accuracy is degraded significantly, especially when the problem size is large. This means that supporting a fully connected Ising model is highly recommended for dealing with a wide range of problems. Another problem is the rapidly increasing connectivity when considering large-scale systems, which usually results in huge energy consumption and latency. Since the number of spins that a particular annealing processor can handle limit the scale of the problem that can be solved, how to solve complex problems with limited hardware in an energy-efficient way has also drawn significant attention.

In this work, we experimentally report a scalable Ising computer based on 80 SMTJs with all-to-all connections and successfully solve the 4761-node TSP problem. The intrinsic stochasticity in SMTJ enables ultra-fast and low-power Ising annealing without using extra resources for random number generation and Metropolis determining process^7^. By combining global annealing with intrinsic annealing in SMTJ, the convergence of the Ising problem is guaranteed especially in large-scale Ising problems. The method to determine parameters of global annealing is discussed. With an all-to-all connection among Ising spins, the combinatorial optimization of 9-city TSP is solved with the optimal solution. We further develop the algorithm for constrained TSP (CTSP) with no extra auxiliary Ising bits both in algorithm and hardware, indicating the superiority and flexibility of this Ising computer. Furthermore, we propose an optimization strategy based on graph partitioning (GP) and CTSP and experimentally solved a 70-city TSP, which typically needs 4761 nodes, on our 80-node Ising computer with a near-optimal solution. The system can obtain the lowest power consumption of 0.64 mW as well as high energy efficiency of 39 solutions per second per watt among state-of-art Ising annealers. We have experimentally demonstrated that large-scale Ising problems can be solved by small-scale hardware in an energy-efficient way.

## Results

### SMTJ-based artificial Ising spin

**Fig. 1:**
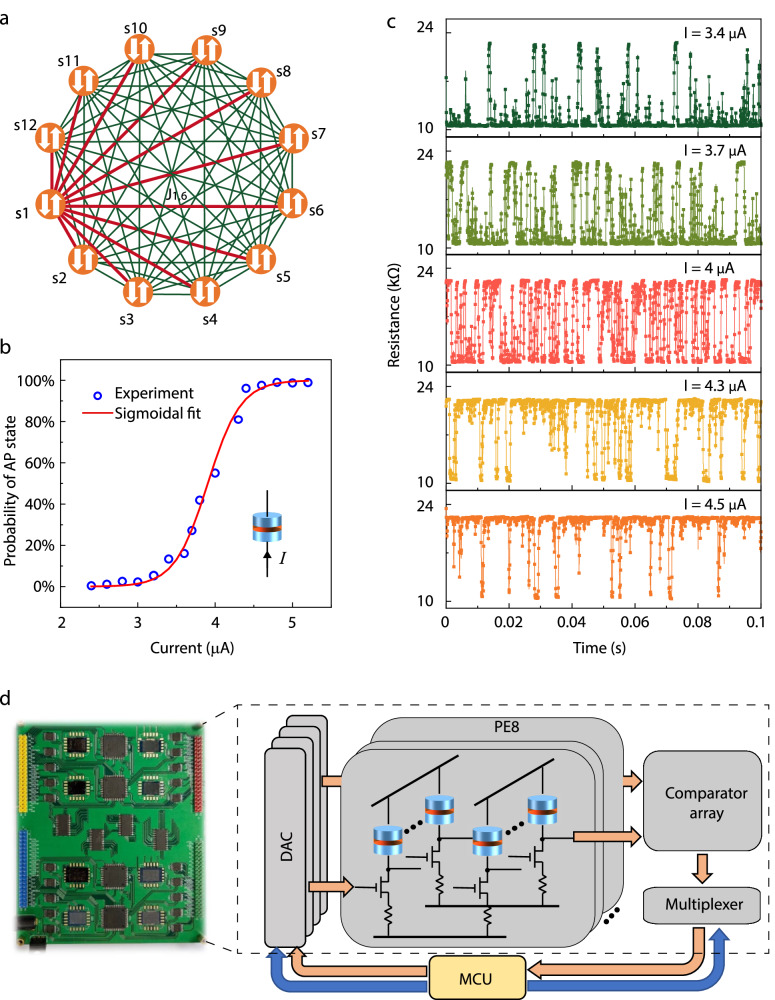
Illustration of SMTJ-based Ising computer. **a** All-to-all connected 12-spin Ising model with s represents the spin and J_1,6_ represents the coupling between s1 and s6. **b** Sigmoidal fit of probability of AP state (${p}_{{AP}}$) of an SMTJ under different input currents (*I*). ${p}_{{{{{{\rm{AP}}}}}}}=\frac{1}{1+{e}^{-4.672\times (I-3.905{{{{{\rm{\mu }}}}}}{{{{{\rm{A}}}}}})}}$. Inset: diagram of an SMTJ. A tunneling barrier layer is sandwiched by a reference layer and a free layer. **c** Time-dependent resistance of an SMTJ under different input currents (*I*). **d** Photograph and schematic diagram of SMTJ-based Ising computer. The system contains 8 processing elements (PEs), 4 digital-to-analog converters (DACs), a comparator array, a multiplexer and a microcontroller unit (MCU). Each PE has 10 SMTJ computing units. Each computing unit includes a transistor and a resistor to adjust the property into stochastic. Blue lines and orange arrows represent the control and data flow, respectively.

Various NP-hard problems can be solved by constructing corresponding Ising models and observing the ground states during evolution processes. Figure 1a shows an all-to-all connected Ising model, whose Ising Hamiltonian can be written as

1 $$H=-{\sum }_{i,j}^{N}{J}_{i,j}{s}_{i}{s}_{j}-{\sum }_{i}^{N}{h}_{i}{s}_{i},$$

where $H$ is the total energy of the system, $N$ is the total number of spins, ${s}_{i}$ is the$\,i$-th spin with one of two states; “+1” (Ising spin up) or “−1” (Ising spin down), ${J}_{i,j}$ is the coefficient of coupling between the $i$-th and the $j$-th spins, and ${h}_{i}$ is the external field of the$\,i$-th spin. For a fixed configuration of other spins than ${s}_{k}$, the probability of ${s}_{k}$ staying in the down-state is given by

2 $${p}{\_}\!\!{{{{{{\rm{\_}}}}}}\downarrow }=\frac{1}{1+{e}^{-2\Lambda }}.$$

where $\Lambda=\frac{\partial H}{\partial {s}_{k}}$ (see Supplementary Note 1).

One natural implementation of this Ising spin is based on a stochastic nanomagnet. The inset of Fig. 1b shows the sketch of an SMTJ, consisting of a tunneling barrier sandwiched by a reference layer and a free layer (see Methods section). Because of the small device diameter (~50 nm), the energy barrier of the free layer between the anti-parallel (AP) and parallel (P) states is low that the retention time of either state is in the range of μs to ms, similar to previous studies^4,35^. The SMTJ resistance, measured as a function of time in Fig. 1c, shows preferred AP states at high currents and P states at low currents. When the current (*I*) is ~4 μA, SMTJ shows an equal chance of AP and P states. The probability of the AP state under different input currents over 0.1 s is fitted in Fig. 1b by a sigmoid function:

3 $${p}{\_}{{{{{{\rm{\_}}}}}}{{{{{\rm{AP}}}}}}}=\frac{1}{1+{e}^{-{{{{{\rm{a}}}}}}\times (I-{{{{{\rm{b}}}}}})}}$$

where ${{{{{\rm{a}}}}}}=4.67 \, {{{{{\rm{and\; b}}}}}}=3.9 \, {{{{{\rm{\mu }}}}}}{{{{{\rm{A}}}}}}.$ In order to emulate Ising spin ${s}_{k}$ with our SMTJ device, we only need to make the probability of the down-state of ${s}_{k}$ to be equal to that for the AP state of SMTJ, namely ${p}{\_}{{{{{{\rm{\_}}}}}}{{{{{\rm{AP}}}}}}}={p}{\_}{{{{{{\rm{\_}}}}}}\downarrow }$, with two calibration coefficients. Thus, we can derive the form of the current ${{I\_}}_{k}$ injected to SMTJ as (see Supplementary Note 1):

4 $${{I\_}}_{k}=\frac{2\Lambda }{a}+b=\frac{c}{a}\left({\sum}_{j}2{J}_{{kj}}{s}_{j}+{h}_{k}\right)+b$$

where $c=1/{kT}$ is the effective inverse temperature which can be conducted for global annealing.

### Intrinsic annealing in SMTJs-based Ising computer

By integrating 80 SMTJs with a peripheral circuit and a microcontroller unit (MCU), we build an 80-node Ising computer (see Supplementary Note 2). Each Ising spin in Eq. (1) is emulated by an SMTJ with intrinsic randomness, where P (AP) state represents spin-up (down). Figure 1d shows the photograph of the printed circuit board (PCB) and the diagram of the system (see Methods section). The system contains 8 processing elements (PEs); each PE has 10 SMTJ computing units. Each SMTJ computing unit includes a transistor and a resistor to adjust the state of SMTJ into stochastic. During the computing process, an MCU examines the states of all SMTJs by reading the output of comparator arrays through multiplexers and generates new input voltages for digital-to-analog converters (DACs) according to the updating rule in Eq. (4) (see Supplementary Note 3 for calibration of 80 SMTJ computing units).

During the evolution process, an Ising solver could be easily trapped in a local minimum state. To avoid this non-optimal solution, annealing algorithms such as simulated annealing (SA) or quantum annealing (QA) were developed. The general idea of SA is to make the system evolve from a high temperature to a low temperature gradually^7^. The convergence and relaxation of SA can be mathematically provable^36^. During each iteration, a random number is generated for stochasticity and introduced to determine whether the result in this iteration should be accepted or not. In QA, quantum fluctuations cause quantum tunneling between states^17^. In both SA and QA, stochasticity needs to be introduced into the annealing process. In contrast, our Ising system utilizes the intrinsic stochastic behaviors of SMTJ to perform the Metropolis process of standard SA in hardware, which greatly saves the solution time and hardware resources for generating randomness (see Supplementary Note 4). Besides, our Ising computer has an all-to-all connection which has wider application scenarios, as well as a better capability of escaping from local minima.

### Ising mapping of N-city TSP and CTSP

**Fig. 2:**
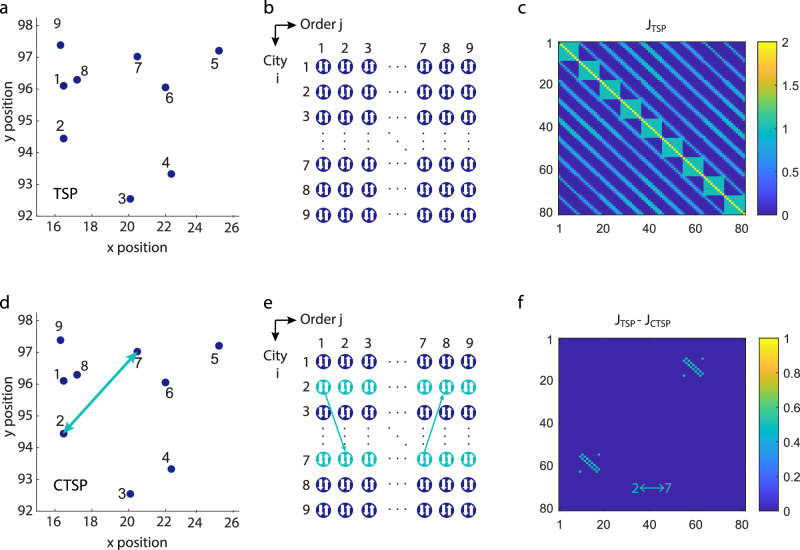
Construct Ising model for a 9-city TSP. **a** Coordinates of all 9 cities used in this problem which are the first 9 cities in the dataset Burma14 from TSPLIB. **b** Ising spin representation for 9-city TSP (81 spins). Rows indicate names of cities and columns indicate the visiting order. Each spin can be 1 (visited) or −1 (not visited) in each iteration. **c** Color map of the coupling matrix J_TSP_ of 9-city TSP, and the color bar represents an effective energy with the unit of *kT*. Here, *k* is the Boltzmann constant and *T* is the temperature. **d** Constrained TSP (CTSP) with a fixed vising sequence from city 2 to city 7 or from city 7 to city 2. The arrows represent the visiting sequence. **e** The Ising spin representation for CTSP with the fixed visiting sequence in **d**. Arrows represent possible vising sequences. **f** Color map of the difference of coupling matrix between TSP (J_TSP_) in **a** and CTSP (J_CTSP_) in **d**. Arrows represent the fixed vising sequences from city 2 to city 7 or from city 7 to 2.

We have applied our Ising computer to the TSP problem, one of the combinatorial optimization problems, which applies to various sectors, such as vehicle routing, logistics, planning, and scheduling. The goal is to find the shortest route that visits all listed cities once and only once given distances between the cities in the list. In order to solve this problem, we first map *N*-city-TSP to an ${N}^{2}$-spin Ising model, or ${(N-1)}^{2}$-spin model assuming a fixed starting city. Figure 2a shows the coordinates of 9 cities and Fig. 2b shows the 81-spin Ising model, whose rows indicate the cities and columns indicate the visiting order. We define the binary spin, *s*, as ${s}_{i,j}$ = 1 if city *i* is visited as *j*-th city or ${s}_{i,j}$ = −1 otherwise. The total Hamiltonian of TSP is expressed by^9^

5 $${H}_{{TSP}}=	 {\sum }_{i}{\left({\sum }_{j}{s}_{{ij}}+(N-2)\right)}^{2}+{\sum }_{j}{\left({\sum }_{i}{s}_{{ij}}+(N-2)\right)}^{2} \\ 	+w{\sum }_{j}{\sum }_{i,i{\prime} }{d}_{i,i{\prime} }\left(\frac{{s}_{i,j}+1}{2}\right)\left(\frac{{s}_{i{\prime},j+1}+1}{2}\right)$$

where the first term is a constraint that represents only one city is visited at the *j*-th visit, and the second term represents one city is visited only one time. $w$ is a constant small enough ($0 \, < \, w \, < \, 1$) not to violate the two constraints of the TSP cycle. ${d}_{i,{i{{\hbox{'}}}}}$ is the distance between city $i$ and city ${i{{\hbox{'}}}}$. According to Eqs. (1) and (5), coupling matrix $J$ of 81 spins could be obtained, as shown in Fig. 2c (see Supplementary Note 5). It shows that spins in the same row or column have strong coupling, as indicated by the first two terms in Eq. (5).

We define CTSP as the visiting orders of some cities are enforced during the traveling. This is quite useful in real-life scenarios. For example, a delivery man collects food and drinks at shop *A* and must deliver hot drinks to *B* first even though the total cost is higher than optimal. We propose an algorithm for solving CTSP by adding negative “distance” to the Hamiltonian. For example, suppose that city A and city B are required to be connected in the CTSP as city 2 and city 7 shown in Fig. 2d, and then we add the term

6 $${-\theta H}_{{{{{{\rm{c}}}}}}}=-\theta \left({\sum }_{j}{s}_{A,j}{s}_{B,j+1}+{s}_{B,j}{s}_{A,j+1}\right)$$

such that the energy of a path, where city A and city B are connected, is always lowered by $\theta .$ When $\theta$ is sufficiently large, the optimal path must have city 2 and city 7 connected. Thus, the total Hamiltonian of the CTSP is expressed by

7 $${H}_{{CTSP}}={H}_{{TSP}}{-\theta H}_{{{{{{\rm{c}}}}}}}.$$

Constructing an Ising model for CTSP is exactly the same as TSP except for extra allowed visiting sequences, as shown in Fig. 2e. This would lead to a modification of the coupling matrix of $J$ according to Eq. (7) (see the deduction of ${J}_{{CTSP}}$ in Supplementary Note 6). From Fig. 2f we can clearly see the differences between ${J}_{{CTSP}}$ and ${J}_{{TSP}}$. This algorithm of CTSP fits for arbitrary constraints of visiting sequences as well as their combinations.

### Experimental demonstration of 9-city TSP

**Fig. 3:**
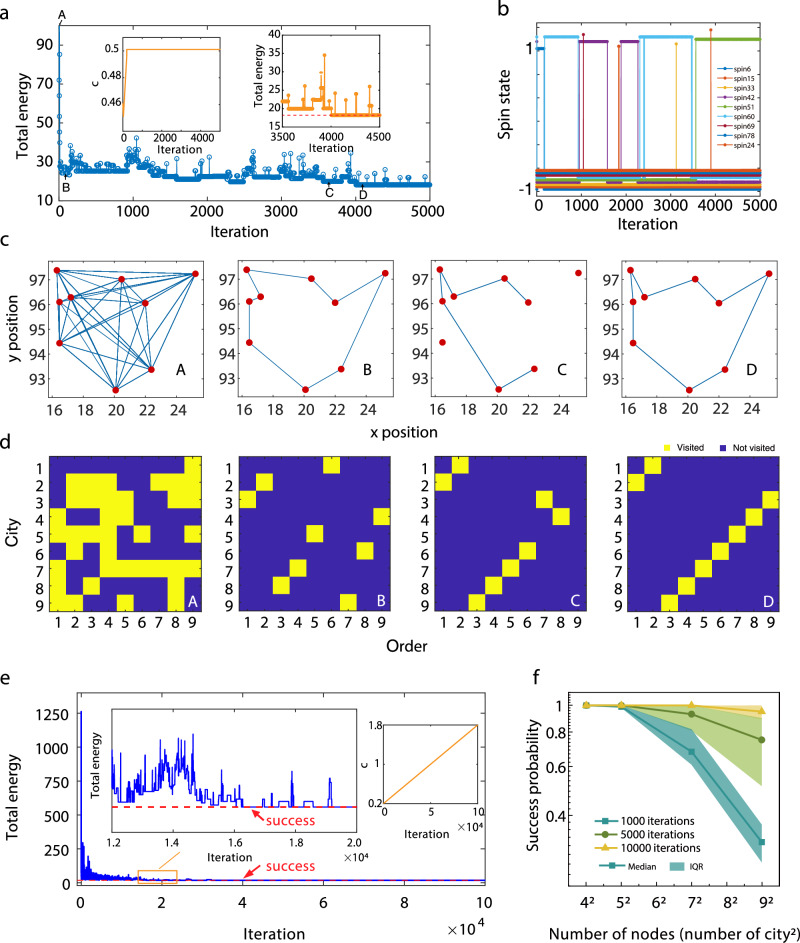
Solution to 9-city TSP. **a** Total energy transition of 9-city TSP with 5000 iterations (the optimal solution with the energy of 18.23 corresponds to the dashed horizontal line). Insets: effective inverse temperature (*c*) and total energy within 3500–4500 iterations. **b** Evolution of 9 representative SMTJ states in 5000 iterations. An offset is used in the *y*-axis to show each SMTJ clearly. **c** Visiting routes of state A, B, C, and D in **a**. **d** Corresponding Ising spins of state A, B, C, and D in **a**. The yellow squares represent ‘visited (${s}_{i,j}=1$)’ and the purple squares represent ‘not visited (${s}_{i,j}=-1$)’. **e** Total energy transition with increasing *c* from 0.2 to 1.8. Left inset: zoom-in view of total energy transition with increasing *c* from 0.392 to 0.52. Right inset: transition of *c* with iterations. The red dashed line represents the optimal path (success). **f** Success probability of solving TSP with varying the node size. The data points and shadows represent the median value and the interquartile range (IQR), respectively.

We first run a 9-city TSP in the 80 SMTJ-based Ising computer at a relatively low but non-zero effective temperature to examine the intrinsic annealing in SMTJ. The iteration time is set comparable to the longest retention time of SMTJs to avoid reading previous spin states. In our experiments, we set the iteration time as 0.1 ms. As shown in Fig. 3a, as the effective inverse temperature (*c*) is increased quickly to 0.5, the system converges rapidly to a low energy state within 50 iterations and reaches the ground state after 4000 iterations. It should be noted that the intrinsic stochasticity in SMTJs helps the system escape from local minima without an extra annealing process, as shown in the right inset of Fig. 3a. Figure 3b illustrates the evolution of 9 spins out of 81 spins. The evolution of all 81 spins can be found in Supplementary Note 7.

We choose four states in Fig. 3a to inspect the traveling path in Fig. 3c and their Ising spins, namely ${s}_{i,j}$, as shown in Fig. 3d. The yellow square in Fig. 3d represents ${s}_{i,j}=1$(visited) and the blue square represents ${s}_{i,j}=-1$(not visited). In an initial state A, the spin states are randomly set and then converge to a relatively low energy at state B. State C is an intermediate solution during the annealing process. State D is the optimal solution satisfying two constraints of the TSP. Because we anneal the system to a relatively low but non-zero temperature so that the convergence to a sub-optimal state could be guaranteed, and at the same time, the intrinsic randomness in SMTJ helps the system to escape from local minima and find a ground state quickly. We test 10 different random initial states each with 5000 iterations and find that in all cases the system can obtain a relatively small energy, as shown in Supplementary Note 8. However, there is a probability that the system jumps out of the ground state because of the non-zero temperature. If we continue to observe the evolution in a large timescale, the system would move back to the global minimum state. In some cases, where the speed and near-optimal solution matter but the accurate optimal solution is not, the number of iterations can be chosen to be small.

Further global annealing of the system to a lower effective temperature may guarantee the convergence of the computation. Here we use linear annealing as an example to examine the convergence of this algorithm in a very large-iteration limit. The initial temperature should be chosen sufficiently high to ensure that the thermal energy exceeds any energy barrier ($\Delta H{=H}_{\max }-{H}_{\min }$) within the system, while still adhering to the fundamental constraints of the specific Ising model. For a given N-city TSP, ${H}_{\max }$ in Eq. (5) can be estimated as $w\times N\times \bar{d}$, assuming that the distance between any two cities is the same as the average distance $\bar{d}$. Similarly, ${H}_{\min }$ can be estimated as $w\times N\times {d}_{\min }$. Therefore, the initial $c$ of 9-city TSP in our experiment can be estimated as ${c}_{{{{{{\rm{initial}}}}}}}\, \sim 1/\Delta H=0.07$, where $w=0.5$, $N=9$ for a total of 9 cities, $\bar{d}=4$ and ${d}_{\min }=0.8$ for the average and shortest distance of each two cities, respectively in Fig. 3c. We then choose ${c}_{{{{{{\rm{initial}}}}}}}$ = 0.2 which is sufficiently safe for annealing. As the temperature linearly decreases, the dynamical system gradually stabilizes. The final temperature should be low enough i.e., ${c}_{{{{{{\rm{final}}}}}}} \, \gg \, 1/\Delta H$, to freeze all possible fluctuations. Here we set ${c}_{{{{{{\rm{final}}}}}}}=1.8$ which is at least one order larger than $1/\Delta H$. This can also be verified by observing randomly generated states under ${c}_{{{{{{\rm{final}}}}}}}$ for long iterations. Regarding the annealing speed, if several changes in the spin configuration are observed under each value of *c*, then this annealing speed is valid. Plenty trials are required to find the proper annealing speed (details in Supplementary Note 8).

In Fig. 3e we can find the first global minimum energy appears after 16,500 iterations, and converge to the ground state after 40,000 iterations. Temperature schedules can be optimized to reduce iteration numbers, e.g. increase the effective temperature in the first few time steps, and then decrease gradually, or learned by the reinforcement learning method^37^. In practice, we use one memory to store the minimum energy state during the computation, and another memory to record the final energy state. We take the minimum value of these two results as the solution. Figure 3f shows the success probability (defined as finding the optimal path) of TSP with various node sizes. The success probability of 9-city TSP reaches 95% after 10^4^ iterations. The success probability with the parameter $w$ in Eq. (5) which determines the relative strength of the constrain term and distance term is also discussed. If the $w$ is too large, then the probabilities of violations, namely the invalid path, would increase, as shown in Supplementary Note 8. If $w$ is too small, then the effect of the distance term is small, which results in a slower convergence to the ground state.

The advantages of this annealer are threefold: (1) Selective working modes by using different temperature schemes. One is the probabilistic sampling mode working at a constant temperature, which is similar to an asynchronous probabilistic computer^4^; the other is the annealing mode conducted by reducing the effective temperature. (2) Fast speed and low power consumption to find the ground state because of the intrinsic annealing properties in SMTJ. (3) Global annealing outperforms probabilistic sampling in achieving efficient convergence, especially for large-scale problems.

We have implemented a synchronous design with a lower requirement on the speed of peripheral circuits. This design also effectively mitigates issues such as leakage, sneak currents, and parasitic resistances which might encountered in asynchronous hardware with a memristive (or resistive) crossbar array.

### Compressing 70-city TSP to 80-node Ising computer

**Fig. 4:**
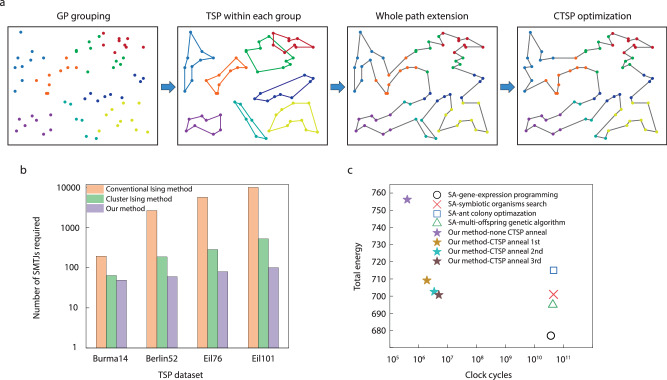
Experimental solution of 70-city TSP. **a** Optimization algorithm for 70-city TSP. **b** Number of required SMTJs for various problems using different methods. Burma14, berlin52, eil76, and eil101 are TSP of 14, 52, 76, and 101 cities, respectively. **c** Comparison of total Ising energy (path) and total clock cycles for final solution with different SA-based algorithms, including symbiotic organisms search^40^, ant colony optimazation^41^, multi-offspring genetic algorithm^42^, and gene-expression programming^7^. Our method is tested on our Ising system and others are tested on Intel Core-i7 PC. In this comparison, our system runs at a main frequency of 10 kHz.

Generally, the number of spins required for an *N*-city TSP is (*N*-1)^2^, which limits the scalability of TSP on state-of-the-art computing systems. Here, we propose a graph Ising compressing algorithm based on CTSP that can significantly reduce the number of spins and interactions for solving a TSP. Figure 4a is an example of how we apply this algorithm to our 80-node SMTJ Ising computer for solving a 70-city TSP (4761 nodes, st70 data set from TSPLIB^38^). The major steps of this algorithm can be described as follows: (a) divide the cities into several smaller groups until the number of cities in each group is less than 10 by GP method; (b) solve TSP within each group separately; (c) integrate neighboring groups to obtain an initial path of the whole group; and (d) optimize the path in (c) by a CTSP window sliding over the whole map.

It is worth mentioning that GP is also an Ising problem. When converting a global TSP into local TSPs, using GP would be more hardware-friendly for our Ising computer compared to other clustering algorithms. It is based on the idea that the original graph can be separated into multiple sub-graphs depending on the Euclidean distance. The number of spins required for solving GP is ~*N* and thus, GP is quite efficient for local TSPs since the problem size can be reduced to ~ ${\left(N-1\right)}^{2}/a$, where $a$ is the number of groups, and each TSP can be optimized independently (see GP mapping in Supplementary Note 9).

The final step (d) is based on CTSP, where a rectangular window slides over the path and cuts it into several disconnected lines, among which the two longest lines are chosen and the edge cities are connected as a circular path (Supplementary Note 10). The CTSP is solved within each window for sub-area optimization without changing the visiting order of edge cities. After this, the two lines at the edge cities are opened and CTSP is carried out again after sliding to the next window. GP-CTSP-based optimization algorithm provides an efficient way of finding near-optimal solutions for large-scale TSP on limited hardware resources.

Figure 4b shows the comparison of numbers of spins for different TSPs by a conventional Ising method^9^, cluster Ising method^39^, and our method. The required number of spins in our method is relatively unchanged for various TSPs, while that of other methods increases substantially with the scale of the problem. Figure 4c shows the total path of 70-city TSP as a function of iteration number using different SA-based algorithms, including symbiotic organisms search^40^, ant colony optimization^41^, multi-offspring genetic algorithm^42^, and gene-expression programming^7^. Finally, we obtain the near-optimal path with a total energy of 700.71, which is slightly higher than the optimal solution of 675. However, the iteration number for an optimized solution is 4.9$\times$10^6^ by our method, which is two to three orders lower than that of SA-based algorithms running on Intel Core-i7 CPU^7^ with the main frequency of 3 GHz, as shown in Fig. 4c.

### Ising computer scaling and cross-bar architecture

The above experimental demonstration shows our Ising computer with 80 SMTJs is capable of finding a near-optimal solution to a medium-scale NP-hard problem. We then explore the performance with increasing from 70 to 200 cities. The simulation of complete TSP task is carried out using MATLAB, incorporating a stochastic model of the SMTJ employed in our experiment (details in Supplementary Note 11). The solution quality is defined as

8 $${Solution}\,{quality}=1-\frac{{total}\,{path}-{best}\,{demonstrated}\,{solution}}{{best}\,{demonstrated}\,{solution}}.$$

**Fig. 5:**
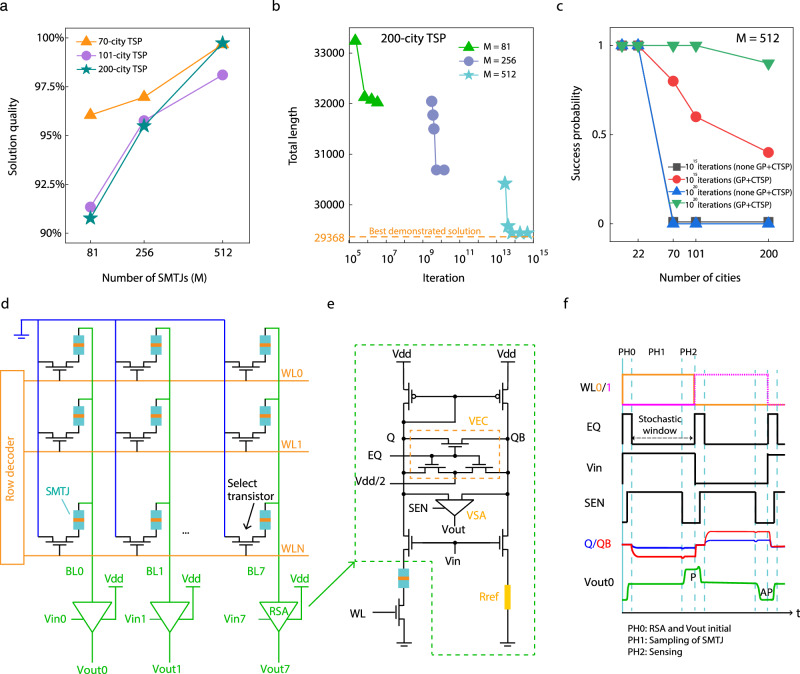
Scaling with problem size and cross-bar implementation towards large-scale Ising computing. **a** Solution quality of various problems using different number of SMTJs (M) in the array. The datasets used are St70, Eil101 and KroA200, for 70, 101 and 200 cities, respectively. **b** Total length of KroA200 TSP at different convergence speeds using different number of SMTJs. The dashed line represents the best demonstrated solution. **c** Success probability of different TSP algorithm (without/with GP and CTSP) as the number of cities increases after running for 50 times. A total of 512 SMTJs are used. Here we define the success as achieving the solution quality of 95%. **d** SMTJ cross-bar array which contains row decoder, SMTJ, select transistor and read sense amplifier (RSA). BL represents bit line, WL represents word line, Vin, Vout and Vdd represent the input voltage, output voltage and supply voltage of RSA. **e** Circuit of one RSA which contains a current mirror, voltage equalization circuit (VEC, with a control signal of EQ which initializes the voltages in Q and QB points, under a reference voltage of Vdd/2), voltage sense amplifier (VSA, with a control signal of SEN), reference resistance (${{{{{\rm{Rref}}}}}}=\frac{1}{2}({{{{{\rm{Rap}}}}}}+{{{{{\rm{Rp}}}}}})$, Rap and Rp represent SMTJ’s resistance in AP and P state respectively), and control transistors. **f** Signals of writing/reading two adjacent SMTJ cells in one BL, selected by WL0 and WL1 in sequence. All signals are defined in **e** and **f**.

Figure 5a illustrates the solution quality of the best results obtained for each TSP task (Supplementary Note 12 for the best solutions). Notably, as the number of SMTJ (M) increases, higher quality solutions can be attained. It is worth emphasizing that the shortest path obtained for the 101-city TSP is 640.9755 in our study, surpassing the optimal path of 642.3095 provided by TSPLIB (Eil101.opt.tour). This outcome serves as evidence of the superiority of our method. The utilization of more SMTJs solving TSP per sliding window leads to improved optimization of CTSP annealing, resulting in an enhanced solution quality, as depicted in Fig. 5b. Consequently, the time to convergence s would also increase with the use of more SMTJS. When dealing with a fixed hardware capacity, an appropriate number of SMTJs for CTSP optimization can be assigned, taking into account both the solution quality and convergence speed. Figure 5c showcases the success rate (defined as achieving 95% solution quality) as the problem size increases. The success probability of 200-city TSP, whose complexity is ~40,000 nodes, can reach as high as 90%, demonstrating the scalability of our method compared to typical TSP (without GP and CTSP)^9^.

We also propose a cross-bar architecture for large-scale Ising computer implementation, which can be integrated by using modern MRAM and CMOS technologies. The core part of this architecture consists of SMTJ bit cells organized as a cross-bar array, integrated with row decoders and read sense amplifiers (RSA), as shown in Fig. 5d. Each SMTJ bit cell contains one select transistor and one SMTJ (1T1SMTJ), whereas the gate of the select transistor is driven by word lines (WL), and the source of all bit cells are connected to the ground. Each bit line is assigned with an RSA. The current flows through SMTJ can be continuously adjusted by Vin of RSA, and the state of SMTJ can be read by RSA at the same time. Figure 5e illustrated the circuit of RSA, in which two clamp transistors control the current flow through the bit cell path and reference path by the gate voltage (Vin), and a current mirror is used to guarantee the same current of the above two paths. Then different voltages would show in the Q and QB point when the resistance of SMTJ is higher or lower than the reference resistor (Rref). By utilizing an enabled voltage sense amplifier (VSA), the voltages at the Q and QB points are sensed, allowing the SMTJ state to be determined as either Vdd (P state) or 0 V (AP state). Particularly, a voltage equalization circuit (VEC) is designed for initializing VSA to avoid incorrect readout. Electrical coupling through a resistance change^43^ is evaluated to have neglectable effects (details in Supplementary Note 11). Figure 5f shows the signals to control and read bit cells. In phase 0 (PH0), one row of SMTJs is selected by WL, and Vin prepared by peripheral circuit is applied to the corresponding RSA. EQ is set high to initialize Q, QB and Vout as Vdd/2. In phase 1 (PH1), the SMTJ fluctuates from the falling edge to next rising edge of EQ. Finally, in phase 2 (PH2), RSAs read the data of one row in parallel at the falling edge of SEN. After the first row has been retrieved, the partial sum starts to be computed. Meanwhile, the same process for the second row can be started, so and so forth. To avoid reading the previous state, the duration of PH1 is preferred to be comparable with the retention time of SMTJ, which limits the main frequency of the system (see details in Supplementary Note 11).

## Discussion

We compare our system with other state-of-art Ising solvers, including CMOS annealer (Intel Core i7 processor)^7^, quantum annealer (D-Wave 2000Q)^16,17^, CIM with FPGA^26^, memristor Hopfield neural networks (mem-HNN)^44^, and phase-transition nano-oscillators (PTNO)^28^ in solving 4761-node TSP70, as shown in Table 1. We use the experimental data for benchmarking from literature, and two kinds of SMTJs for comparison. One is our perpendicular anisotropy SMTJ device and the other is assuming recently reported in-plane anisotropy SMTJ with a retention time of 8 ns^45,46^. The major attributes are the main frequency (defined as 1/iteration time), power, time-to-solution as well as energy efficiency (defined as solutions per second per watt). As quantum computers, CIM, mem-HNN, and PTNO only demonstrated ~100-node max-cut problems, we estimate the time-to-solution for solving TSP70 by assuming that the algorithm and the total number of spins to find a near-optimal solution is the same as our work (details in Supplementary Note 13). Here, we set 80-spin Ising computer as a standard and fix the number of iterations of 400,000 for a good solution to TSP70. Only Ising computing parts are calculated for power consumption.

**Table 1:** Comparison of state-of-art Ising computers

	CPU	Quantum annealers	CIM	Mem-HNN	PTNO	SMTJ-based Ising computer		
Spin form	Artificial Ising spin	Qubits	Optical parametric oscillator	Memristor	Nano-oscillators	MTJ		
Algorithm	Simulated annealing	Quantum annealing	Coherent computing	Modulate intrinsic noise annealing	Simulated annealing	Intrinsic noise annealing + global annealing		
Connectivity	All-to-all	Sparse	All-to-all	All-to-all	All-to-all	All-to-all		
Room temperature	Yes	No	Yes	Yes	Yes	Yes		
Number of nodes solved experimentally (simulated)	4761	61	2000	60	8 (100)	4761		
Number of devices (bits)	~10^9^	2048	1 (2048)^d^	3600	8	80	4096	
Power	65 W	25 kW		120 mW	2.56 mW^h^	Experiment	Simulation using SMTJ in this work	Simulation using SMTJ in ref 45^m^
						0.64 mW^j^	0.288 mW^l^	0.288 mW
Size of the computer (chip)	14 cm^2^		3$\times {10}^{4}$ cm^3^	64 cm^2^		8 cm^2^	12288 µm^2^	12288 µm^2^
Main frequency	3 GHz^a^		200 kHz^e^	1 MHz^g^	301 Hz^i^	10 kHz	10 kHz	1.1 MHz
Time to solution (TSP70)	12 s^b^	>10^4^ s^c^	80 ms^f^	320 ms	1.3 × 10^3 ^s	40 s^k^	50.54 s	0.64 s
Energy to solution (TSP70)	780 J	>2.5$\times$10^8 ^J		3.84$\times$10^−2 ^J	3.4 J	2.56$\times$10^−2^ J	1.456$\times$10^−2^ J	1.8$4\times$10^−4^ J
Energy efficiency (1/energy to solution)	1.3$\times$10^−3^	<4.0$\times$10^−9^		<2.6$\times$10^1^	2.9$\times$10^−1^	3.9$\times$10^1^	6.8$\times$10^1^	5.4$\times$10^3^

Unavailable quantities are left blank.

^a^Estimated average main frequency of Intel Core-i7 PC.

^b^Solution time using dataset st70 from ref. ^7^ by using TSP-SAGEP algorithm.

^c^Approximation from the result of *N* = 55 node MaxCut problem.

^d^1-km fiber with 2048 pulses per second used as Ising spins in a time-multiplexed way.

^e^Round-trip time of 5 µs in experiment.

^f^Linear approximation from the simulated result of *N* = 2000 node complete graph problem. The time of transferring data from FPGA to computer is 60 s×n, where n is the observation time.

^g^Main frequency of on-chip computing array using 180-nm CMOS-memristor chip in experiments.

^h^Estimated power consumption of main compute kernel.

^i^Estimated for 80 node system.

^j^Experimentally demonstrated power consumption of main compute kernel.

^k^Experimentally tested total solution time using dataset st70 from TSPLIB including computing time, data transfer time, and updating time.

^l^Power consumption of main compute kernel of 4 Kb SMTJ Ising computer (4-bit bit line) based on the experimentally calibrated SMTJ model.

^m^Simulation results using SMTJ with a retention time of 8 ns in ref. ^45^.

In Table 1, although the main frequency of CPU is the highest among all candidates, the energy efficiency is lower than our SMTJ-based approach. This is due to the redundant logic and data transfer delay between the memory and PEs in a conventional von-Neumann architecture. The SMTJ-based approach currently outperforms the quantum annealer both in the power consumption as well as time to solution. The power of quantum annealer is huge which needs to be optimized further for real applications. CIM is another promising architecture with a fast speed and acceptable power consumption. Current CIM systems are proof-of-concept systems which are not at present optimized for energy efficiency. Mem-HNN has a relatively fast speed assuming the 180-nm CMOS technology. However, the required number of devices is large, which limits the integrated density. The PTNO approach uses capacitors or resistors to mimic spin coupling, whose main frequency would be limited by the system scale and parasitic effects. It is reported that the ideal main frequency would decrease from 500 to 87 MHz when the system scale increases from 8-node to 100-node^28^. Our SMTJ-based Ising computer outperforms other approaches with low power consumption with 0.64 mW (details in Supplementary Note 13).

We experimentally demonstrate perpendicular MTJs with a retention time of ~0.1 ms and solve TSP70 Ising problems at an energy efficiency of 39 solutions per second per watt. Furthermore, we simulate an Ising computer with 4 Kb SMTJs using 40 nm commercial CMOS technology. The simulated energy efficiency for solving TSP70 by using the same SMTJ can reach 68 solutions per second per watt. By using reported in-plane SMTJ^45^ and advanced CMOS, the system could obtain the highest energy efficiency of $5.4\times {10}^{3}$, which shows several orders of magnitude improvement over other approaches. This result suggests that an SMTJ-based Ising computer can be a good candidate for solving dense Ising problems in a highly energy-efficient and fast way.

In summary, we have experimentally demonstrated an intrinsic all-to-all Ising computer based on 80 SMTJs, and solved 9-city TSP with the optimal solution. Furthermore, a compressing strategy based on CTSP and GP is proposed to experimentally solve 4761-node 70-city TSP on an 80-node system with a near-optimum solution as well as ultra-low energy consumption. A cross-bar architecture is then proposed for large-scale Ising computers and the 200 city TSP task is simulated. Our system provides a feasible solution to fast, energy-efficient, and scalable Ising computing schemes to solve NP-hard problems.

## Methods

### Sample growth and device fabrication

Thin film samples of substrate/[W (3)/Ru (10)]_2_/W (3)/Pt (3)/Co (0.25)/Pt (0.2)/[Co (0.25)/Pt (0.5)]_5_/Co (0.6)/Ru (0.85)/Co (0.6)/Pt (0.2)/Co (0.3)/Pt (0.2)/Co (0.5)/W (0.3)/CoFeB (0.9)/MgO (1.1)/CoFeB (1.5)/Ta (3)/Ru (7)/Ta (5) were deposited via DC (metallic layers) and RF magnetron (MgO layer) sputtering on the Si substrates with thermal oxide of 300 nm with a base pressure of less than $2\times {10}^{-8}$ Torr at room temperature. The numbers in parentheses are thicknesses in nanometers. To fabricate the superparamagnetic tunnel junctions, bottom electrode structures with a width of 10 µm were firstly patterned via photolithography and Ar ion milling. MTJ pillar structures with a diameter of ~50 nm for the superparamagnetic behavior were patterned by using e-beam lithography. The encapsulation layer of Si_3_N_4_ was in-situ deposited after ion milling without breaking vacuum by using RF magnetron sputtering, and top electrode structures with a width of 10 µm were patterned via photolithography and top electrodes of Ta (5 nm)/Cu (40 nm) were deposited by using DC magnetron sputtering.

### MTJ characterization by probe station

The setup includes a source meter (Keithley 2400) for supplying DC bias currents and a data acquisition card (NI-DAQmx USB-6363) for the read operation. A single SMTJ operation cycle comprises two steps (i.e. bias and read). A small DC input current with an amplitude of 1–20 μA is applied to SMTJ. Simultaneously, the DAQ card reads the voltage signal across the SMTJ at a maximum sampling rate of 2 MHz. The MTJ switching probability varies in accordance with the amplitude of applied currents. The retention time of MTJ is determined from random telegraph noise measurements over 250 ms. The expectation values of event time τ is determined by fitting an exponential function to the experimental results.

### Ising PCB

80 SMTJ arrays and peripheral circuits are integrated on a 12 cm × 15 cm PCB, controlled by an MCU (Arduino Mega 2560 Rev3). Four 12-bit rail-to-rail DACs (AD5381) with 160 output channels in total are used to generate analog DC inputs for PE and comparator arrays. Half of the DAC output channels are used to provide stimulation to the gate terminal of NMOSs (2N7002DW-G), and others are used to provide reference voltages to comparators (AD8694). The drain voltages of NMOS are compared with reference voltages and generate outputs in parallel. Outputs of comparator arrays are read by MCU through four multiplexers (FST16233) and then are calculated to obtain new inputs for DACs. The supply voltage of the PCB board and SMTJs is 5 V and 0.8 V, respectively. The value of resistors in each computing unit can be designed to adjust the center of sigmoidal curves.

### Supplementary information

Supplementary Information

### Source data

Source Data

## Data Availability

The data generated during this study are available within the article and the Supplementary Information file. Source data are provided with this paper.

## Code availability

The codes that support this study can be available from the corresponding author upon request.
