# Supplementary material for: Energy-efficient superparamagnetic Ising machine and its application to traveling salesman problems
Source: Nat Commun. 2024 Apr 24;15:3457. doi: 10.1038/s41467-024-47818-z (PMC11043373; doi:10.1038/s41467-024-47818-z)
Supplement: Supplementary file 1 — Supplementary Information [file 41467_2024_47818_MOESM1_ESM.pdf]

Energy-efficient superparamagnetic Ising machine and its application to traveling salesman problems

Jia Si[1,2], Shuhan Yang[1], Yunuo Cen[1], Jiaer Chen[1], Yingna Huang[1], Zhaoyang Yao[1], Dong-Jun Kim[1], Kaiming Cai[1], Jerald Yoo[1], Xuanyao Fong[1] and Hyunsoo Yang[1]*

[1]Department of Electrical and Computer Engineering, National University of Singapore, Singapore, Singapore.

[2]Key Laboratory for the Physics and Chemistry of Nanodevices and Center for Carbon-based Electronics, School of Electronics, Peking University, Beijing, China.

* Corresponding author. Email: eleyang@nus.edu.sg

Supplementary Note 1. Construct artificial Ising spin and form of update current

We first examine the SMTJ properties in our experiments. From the results in Figure. 1b, we can summarize that the probability of P state of SMTJ follows a sigmoidal function with the input current I_k, which can be written as

$$p_{\_P} = \frac{1}{1+e^{I_k}} \tag{1}$$

where $I_k = a(I_{exp} - I_{50\_50})$, a is a non-ideal coefficient varying among SMTJs, I_{exp} is the current injected to SMTJ, I_{50_50} is a bias current, $a > 0$ (a < 0) indicates that positive (I_{exp}– I_{50_50}) favors the P (AP) state.

We then consider the Boltzmann probability distribution of the Ising spin s_k from the TSP model. For a fixed configuration of other spins than s_k, the probability of s_k staying in the up-state is given by

$$p_{\_\uparrow} = \frac{e^{-E[s_{k\uparrow},\{s_{others}\}]/\mathrm{kT}}}{e^{-E[s_{k\uparrow},\{s_{others}\}]/\mathrm{kT}}+e^{-E[s_{k\downarrow},\{s_{others}\}]/\mathrm{kT}}} \tag{2}$$

where $E[s_{k\uparrow},\{s_{others}\}]$ represents the system energy when s_k is spin-up, and $E[s_{k\downarrow},\{s_{others}\}]$ represents the system energy when s_k is spin-down, and $\{s_{others}\}$ represents the configuration of other spins. Here other spins are regarded as a background. Here, *k* is the Boltzmann constant and *T* is the temperature.

We define $\Lambda = \frac{\partial E}{\partial s_k}$, then have $\Lambda = \frac{E[s_{k\uparrow},\{s_{others}\}]-E[s_{k\downarrow},\{s_{others}\}]}{2}$ and derive the explicit form of Λ and the average energy of the system, $E_0 = \frac{E[s_{k\uparrow},\{s_{others}\}]\mp E[s_{k\downarrow},\{s_{others}\}]}{2}$. Thus, the system energy, when s_k is spin-up and spin-down, can be rewritten as, respectively

$$E[s_{k\uparrow},\{s_{others}\}] = E_0 + \Lambda \tag{3}$$

$$E[s_{k\downarrow},\{s_{others}\}] = E_0 - \Lambda. \tag{4}$$

Comparing Supplementary Eq. (3) and Supplementary Eq. (4) with Supplementary Eq. (2), we have

$$p_{\_\uparrow} = \frac{e^{(-\Lambda - E_0)/\mathrm{kT}}}{e^{(-\Lambda - E_0)/\mathrm{kT}} + e^{(\Lambda - E_0)/\mathrm{kT}}} = \frac{1}{1+e^{2\Lambda/\mathrm{kT}}}. \quad (5)$$

If we want to emulate Ising spin s_k with our SMTJ device, we only need to let the probability of up-state of s_k and P state of SMTJ equals to each other, namely $p_{\_P} = p_{\_\uparrow}$. Thus, the updating current of SMTJ can be derived as

$$I_{\_k} = \frac{2\Lambda}{a\mathrm{kT}} + \mathrm{b} = \frac{c}{a}\left(\sum_j 2 J_{kj} s_j + h_k\right) + b \quad (6)$$

where $c = 1/\mathrm{kT}$ is the inverse temperature, h_k is the local magnetic field, and J_{kj} is the coupling coefficient between s_k and s_j. The spin current I_k shares the same form where the current is determined by solving Fokker-Planck description[1].

Supplementary Note 2. Ising computing PCB board

To implement the connections and scale up to 80 spins with SMTJ (8 PEs, 10 SMTJ in each PE), we utilized the measurement-feedback scheme[2,3]. Based on the measurement-feedback scheme, the requirement of hardware resource scales at $O(n)$ and $O(n^2)$ for implementing the Ising bits (including the SMTJs for p-bit, digital to analog converters (DACs) for input, comparators for read-out) and the connections (for the computation in Supplementary Eq. (6)). Supplementary Figure 1 illustrates the photo of Ising computing PCB board and circuits. MCU reads 16 SMTJs in parallel from MUX and after 5 cycles all states of 80 SMTJs are achieved. Then MCU calculates update voltages (Vin) through matrix multiplication based on states of 80 SMTJs, weights (J) and calibration parameters. Instructions carrying update voltages are sent to DAC blocks (DA_ref and DA_data blocks in Supplementary Figure 1c) and the outputs of these DAC blocks are updated accordingly. Above two DA_ref blocks represent DAC modules, which generate reference voltages. DA_data blocks represent the DAC modules, which generate update voltages.

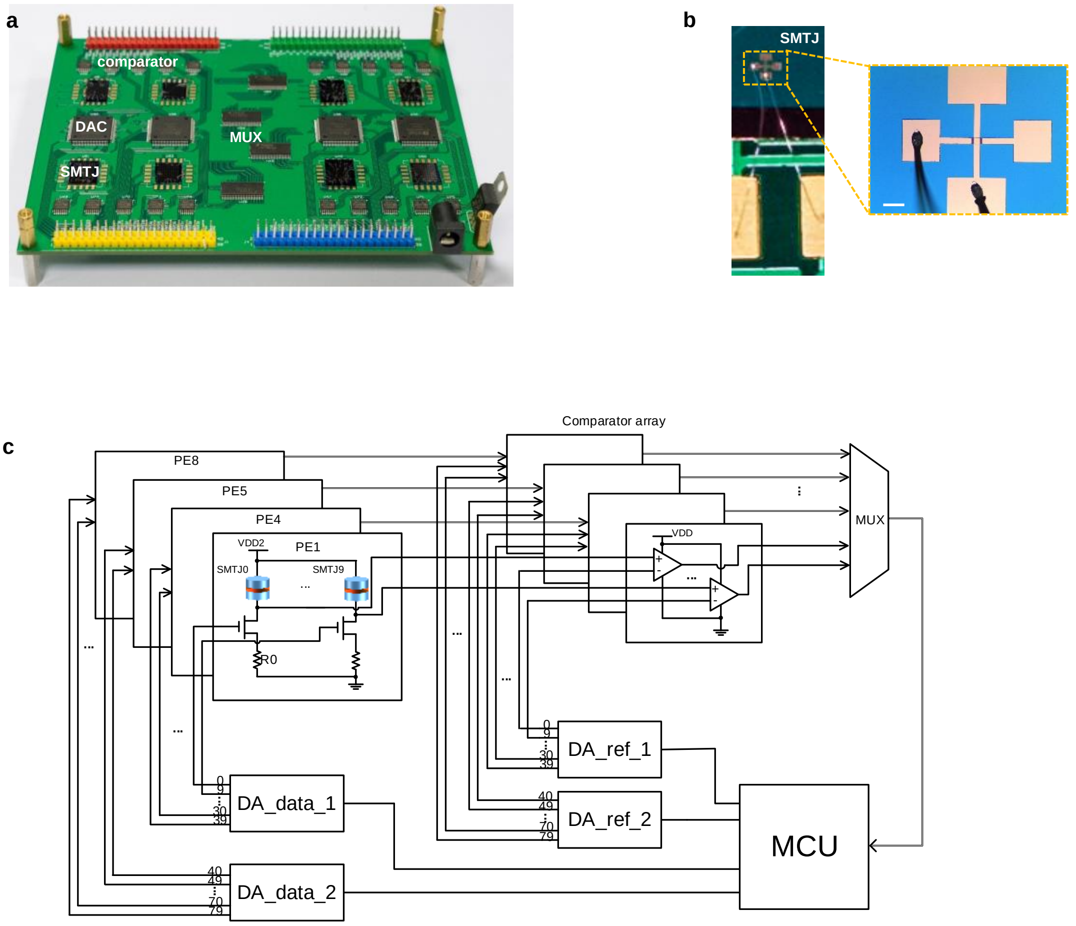

Supplementary Figure 1. Photograph of Ising computing board and circuit of the system. **a**, Ising computing board integrated with 12-bit rail-to-rail DACs (AD5381) with 40 channels, comparators (AD8694), and multiplexers (FST16233). **b**, Zoom-in photograph and microscope image of SMTJ with bonding wires. The scale bar is 100 µm. **c**, Circuit of the system.

Supplementary Note 3. Calibration of SMTJ computing units

We calibrate 80 SMTJ computing units with two steps. I_{exp} represents the current injected to SMTJ. First, we calculate the resistance of $R0$ within each unit to adjust the centre input voltage with 50-50% of AP-P states according to Supplementary Eq. (7)

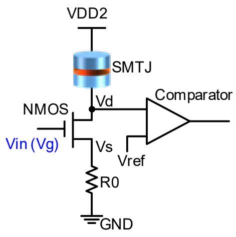

$$V_{DD2} = I_{50\_50} \times \left(\frac{R_P+R_{AP}}{2} + R0\right) + Vds$$

$$Vgs = Vg - Vs$$

$$Vds = Vd - Vs$$

$$R0 = \frac{Vs}{I_{50\_50}} \qquad (7)$$

where V_{DD2} is set as 0.8 V and Vg_{50_50} is around 1.7 V. The results after $R0$-calibration are shown in Supplementary Figure 2b. Second, we fit the results in Supplementary Figure 2b by the sigmoidal equation with two parameters *a'* and *b'*, $p_{\_AP} = \frac{1}{1+e^{a'\times(Vin-b')}}$, and obtain a standard sigmoidal curve in Supplementary Figure 2c**.** We can regard the term $a' \times (Vin - b')$ as the ideal input TSP current I_TSP, and then we have $p_{\_AP} = \frac{1}{1+e^{I\_TSP}}$. Thus, in real Ising calculations such as GP and TSP in our work, we only need to calculate the ideal updating current I_TSP in each iteration from the Ising model, and then transform I_TSP to real updating voltages Vin (Vg) for each SMTJ computing units by parameters *a'* and *b'*

$$Vin = \frac{2}{a'} \times I\_TSP + b'. \qquad (8)$$

Note that these fitting parameters are determined for one time after choosing $R0$ and before Ising computing. For a large system calibration, applying different values of $R0$ in hardware can be replaced by applying the same value of $R0$ and fitting parameter b' from the sigmoidal curve through software. The range of Vin applied to each SMTJ is not a problem as long as the NMOS still works in an effective region because SMTJ is individually programmed. Besides, the stochastic system is highly fault-tolerant and not sensitive to $R0$. For example, the resistance of SMTJ is quite large (10 ~ 20 kΩ), and thus the precision of $R0$ only needs to be ~

kΩ. As for time consumption, SMTJs can be divided into several groups and calibrated in parallel.

The resistance dispersion of the AP and P states should be also considered to achieve the correct electrical readout of the SMTJ states. Supplementary Figure 3a illustrates the actual resistance dispersion, from which we can observe there is a window between AP state and P state. Note that the resistance value we used in the calibration process already contained information on finite bias-dependent TMR reduction. In our case, the effective working current of SMTJ was very small, as shown in Supplementary Figure 2a, thus the reading voltage for SMTJ was small. Supplementary Figure 3b shows the typical R-V curve of an SMTJ, from which we can find that the effective working window defines a maximum reading voltage of 0.15 V. Under this small reading voltage, the TMR ratio slightly reduced from 102.88% to 92.66%, where the AP state and P state can still be distinguished easily. The value of reference voltage V_{ref} is set as $V_{\mathrm{ref}} = V_{\mathrm{DD2}} - \frac{R_{\mathrm{AP}}+R_{\mathrm{P}}}{2} \times I_{\mathrm{exp}}$, where I_{exp} can be calculate by $I_{\mathrm{exp}} = \frac{a' \times (Vin-b')}{c'} + d'$. The parameters c' and d' is obtained by fitting the results in Supplementary Figure 2a as $p_{\_AP} = \frac{1}{1+e^{c' \times (Iexp-d')}}$ before Ising computing. Supplementary Figure 4 shows the voltage-time trace of the comparator block with a typical computing unit of $R_{\mathrm{AP}} = 22.5\ k\Omega$, $R_{\mathrm{P}} = 12.6\ k\Omega$, and $R_0 = 80\ k\Omega$. Note that V_{ref} could also be fixed if SMTJ satisfies $\frac{R_{\mathrm{AP}}}{R_{\mathrm{P}}} > \frac{I_{\mathrm{exp\_max}}}{I_{\mathrm{exp\_min}}}$, and then the value of V_{ref} can be set as $V_{\mathrm{ref}} = V_{\mathrm{DD2}} - \frac{I_{\mathrm{exp\_max}} \times R_{\mathrm{P}} + I_{\mathrm{exp\_min}} \times R_{\mathrm{AP}}}{2}$.

The retention time of SMTJ is determined from random telegraph noise (RTN) measurements using the setup in Supplementary Figure 5a. Supplementary Figure 5b shows an example of the histogram of the event determined from RTN measurements over 250 ms. The number of the event time N shows a typical exponential distribution $N = \frac{1}{\tau} exp(-\frac{t_{event}}{\tau})$, indicating the switching event follows a Poisson process. The expectation values of event time τ is determined by fitting an exponential function to the experimental result shown in the solid

lines in Supplementary Figure 5b. We define the average retention time $\tau = \sqrt{\tau_{AP} \times \tau_P} \approx$ 0.123 ms.

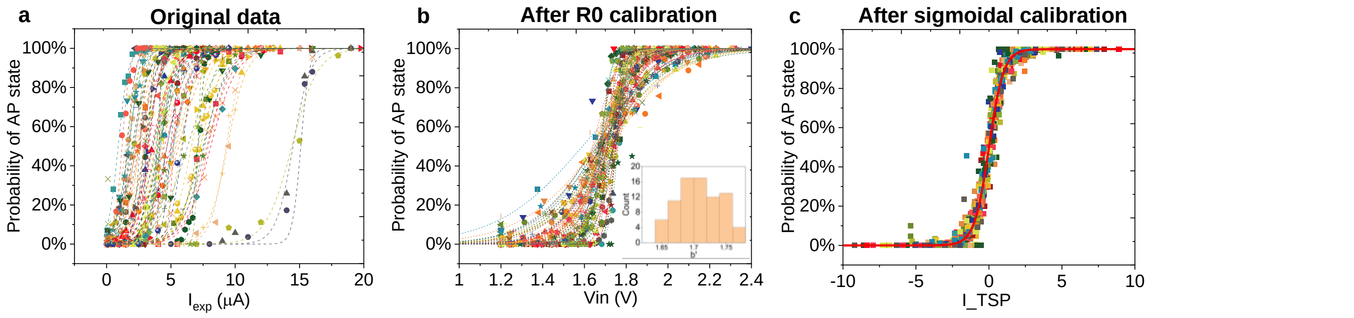

Supplementary Figure 2. Calibration of SMTJ computing units. **a**, Original SMTJ data measured by data acquisition card (DAQ card). **b**, Calibration after choosing proper *R*0 for each SMTJ. **c**, Further calibration by the sigmoidal function with *a* and *b* parameters.

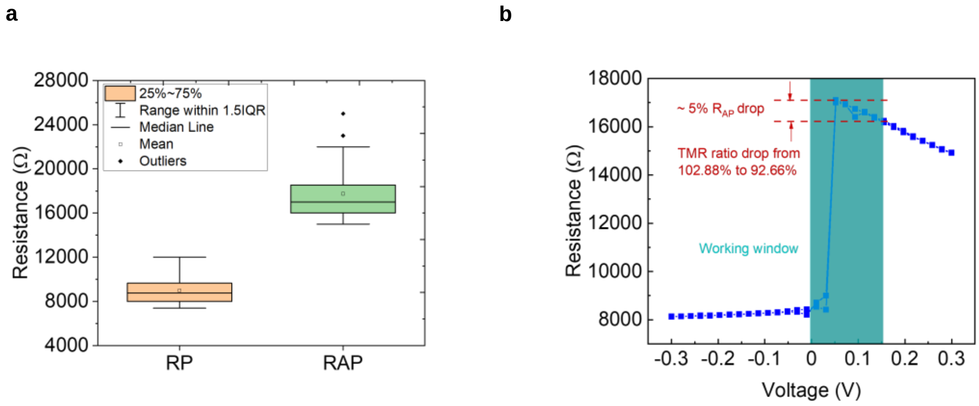

Supplementary Figure 3. Resistance dispersion and the bias-dependent TMR reduction of SMTJ. **a**, Resistance dispersion of P and AP states. **b**, Bias-dependent TMR reduction of an individual SMTJ.

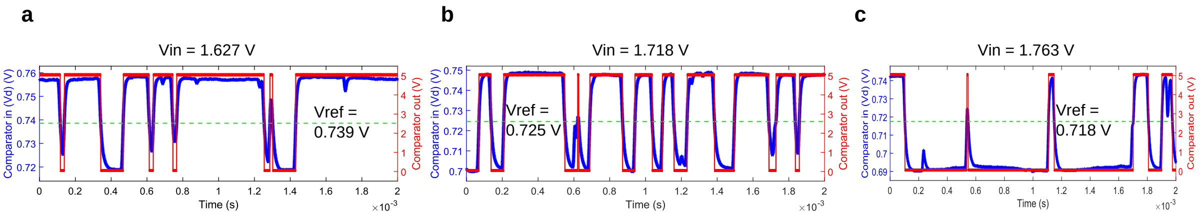

Supplementary Figure 4. Voltage-time trace of the comparator block under different Vin. **a**, Vin = 1.627 V. **b**, Vin = 1.718 V. **c**, Vin = 1.763 V.

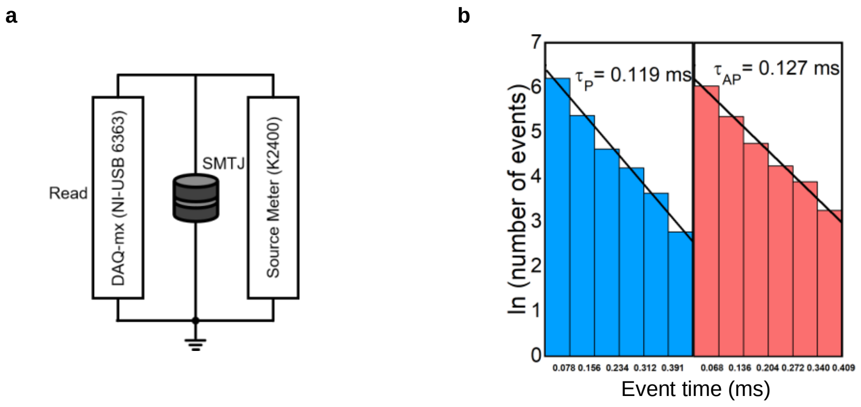

Supplementary Figure 5. RTN measurements. **a**, Experimental setup. **b**, Histogram of the event time τ for P and AP states.

Supplementary Note 4. Flowchart of SA and SMTJ-based Ising computing system

a

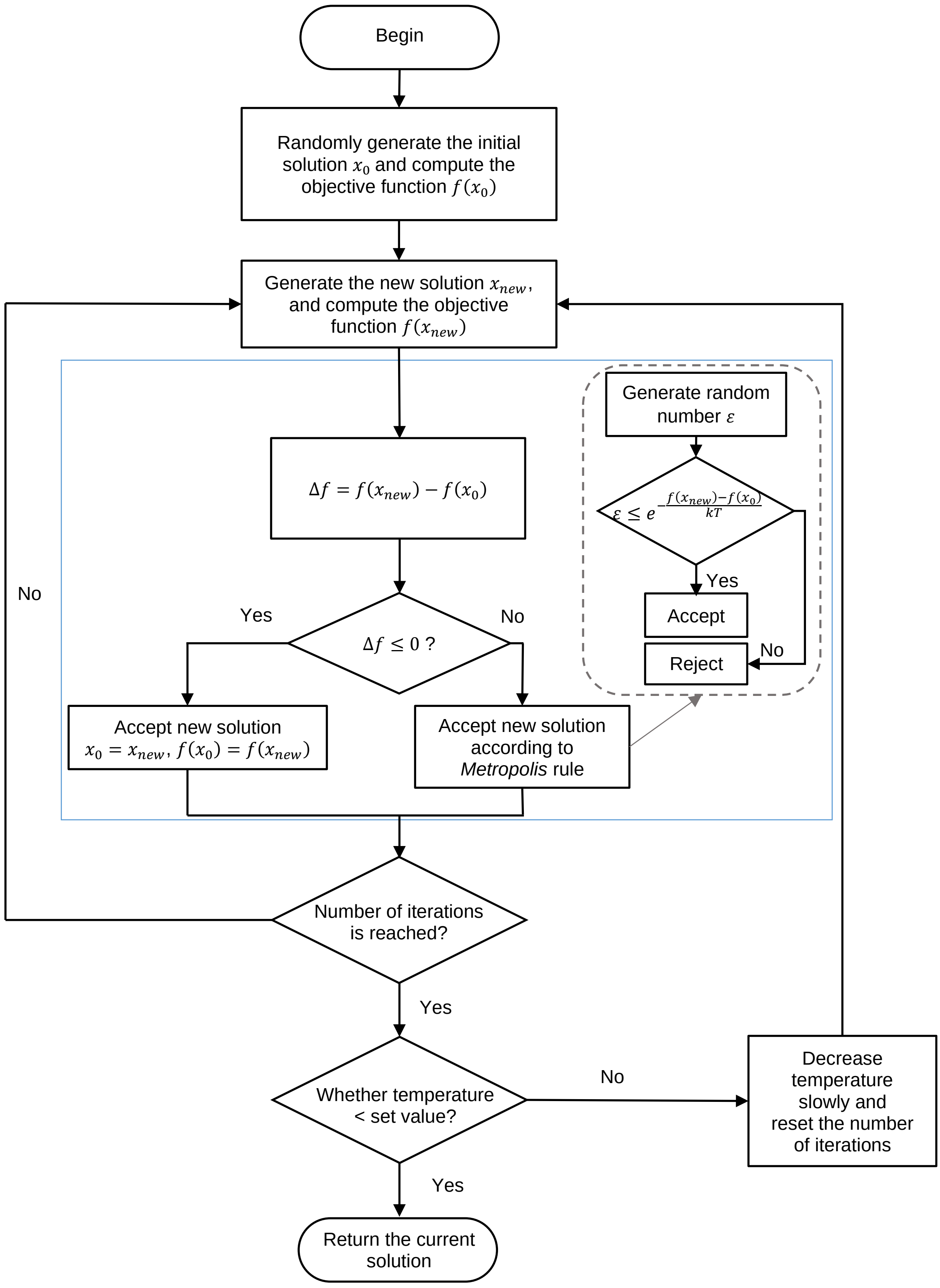

b

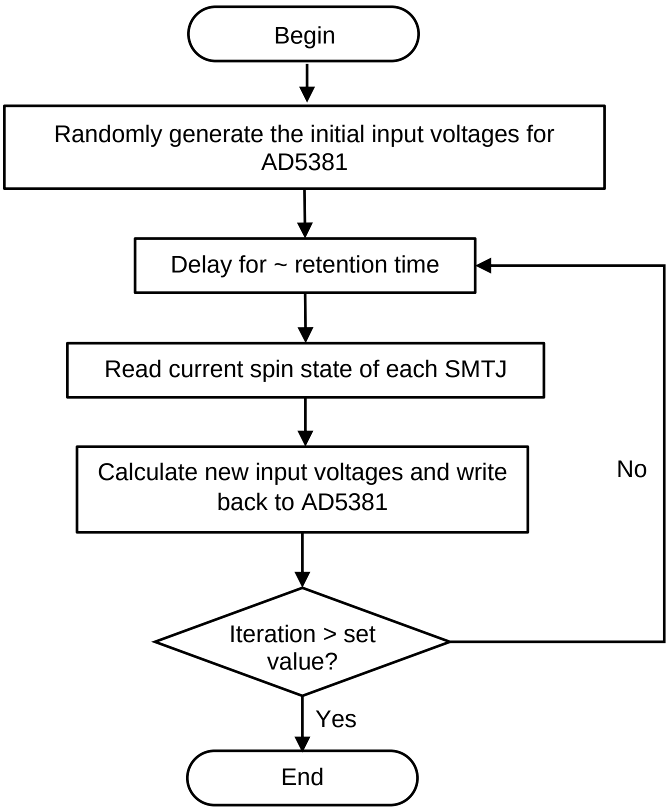

Supplementary Figure 6. Flowcharts of standard SA and Ising computing process. a, Flowchart of SA[4]. The objective function is the total energy of the path. Steps in standard SA indicated by a blue box can be achieved by hardware in SMTJ-based Ising computing system. **b**, Flowchart of Ising computing system based on SMTJs. AD5381 is a DAC which converts the digital data to analog voltages.

Supplementary Note 5. Mapping rules of Ising Hamiltonian of TSP (circle path)

We use 1 to represent spin-up and –1 to represent spin-down. Thus the constrain term of TSP is expressed by[5]

$$H_A = \lambda_A \sum_i (\sum_j s_{ij} + (N-2))^2 + \lambda_A \sum_j (\sum_i s_{ij} + (N-2))^2$$

$$= \lambda_A \sum_i \left(\sum_{j_1,j_2} s_{ij_1} s_{ij_2} + 2(N-2) \sum_j s_{ij} + (N-2)^2 \right)$$

$$+ \lambda_A \sum_j \left(\sum_{i_1,i_2} s_{i_1 j} s_{i_2 j} + 2(N-2) \sum_i s_{ij} + (N-2)^2 \right)$$

$$= \lambda_A \textstyle\sum_{ij_1j_2} s_{ij_1} s_{ij_2} + \lambda_A \sum_{i_1i_2j} s_{i_1j} s_{i_2j} + 4(N-2)\, \lambda_A \sum_{i,j} s_{ij}. \quad (9)$$

The first term is a constraint that represents a penalty for visiting multiple vertices as the j-th visit, which takes a minimum value of 0 when only one city is visited. The second term is also a constraint that represents one city is visited only one time.

The total energy of the path, which is the objective function (distance term) is expressed as

$$H_B = \lambda_B \sum_j \sum_{i,i'} d_{i,i'} \left(\frac{s_{i,j} + 1}{2} \right) \left(\frac{s_{i',j+1} + 1}{2} \right)$$

$$= \tfrac{\lambda_B}{4} \textstyle\sum_j \sum_{i,i'} d_{i,i'} \big(s_{i,j}\, s_{i',j+1} + s_{i,j} + s_{i',j+1} + 1 \big). \quad (10)$$

Thus, the total Hamilton of TSP can be calculated as $H = H_A + WH_B$ where W is a coefficient number, which is small enough that it is never favourable to violate the constrain terms of H_A. Finally, we obtain the total Hamiltonian for circle path TSP

$$H = \sum_{ij_1j_2} s_{ij_1} s_{ij_2} + \sum_{i_1i_2j} s_{i_1,j} s_{i_2,j} + \frac{w}{4} \sum_{j,i,i'} d_{i,i'} s_{i,j} s_{i',j+1}$$

$$+ 4(N-2)\lambda_A \sum_{i,j} s_{i,j} + \frac{w}{4} \sum_{j,i,i'} s_{i,j} d_{i,i'} + \frac{w}{4} \sum_{j,i',i} s_{i',j+1} d_{i,i'}\,.$$

(11)

If we compare it with the definition equation of Hamiltonian, $H = \sum_{iji'j'} J_{ij,i'j'} s_{ij} s_{i'j'} + \sum_{i,j} h_{ij} s_{ij}$, we can deduce the form of correlation matrix J and local magnetic field h as follows.

$$\begin{cases} J_{ij,i'j'} = \delta_{i,i'} + \delta_{j,j'} + \frac{w}{4}(\delta_{j,j'-1}\, d_{i,i'}) = \delta_{i,i'} + \delta_{j,j'} + \frac{w}{4}(\frac{1}{2}(\delta_{j,j'-1}\, d_{i,i'} + \delta_{j,j'+1}\, d_{i,i'})) \\ h_{ij} = 4(N-2)\lambda_A + \frac{w}{2}\sum_{i'} d_{i,i'} \end{cases}$$

(12)

Supplementary Note 6. Mapping rules of Ising Hamiltonian of constrained TSP (CTSP)

We first propose the algorithm for constrained TSP, *i.e.* to fix the sub visiting sequence of two or more cities during the path. For example, TSP with constraints of C&D means if the traveller is in city C, then one must visit city D next, or if one is in city D then one should visit city C next.

In this case, we should add one more term in Hamiltonian as a constrain, $Hc = \sum_j s_{C,j}s_{D,j+1} + s_{D,j}s_{C,j+1}$, therefore the total Hamiltonian of this problem is $H = H_A + wH_B - \theta Hc$.

We choose $\theta \approx 1$ to guarantee constraints of H_A and Hc, and then we can deduce the form of J and h. Comparing

$$H = \sum_{ij_1j_2} s_{ij_1}s_{ij_2} + \sum_{i_1i_2j} s_{i_1,j}s_{i_2,j} + \frac{w}{4}\sum_{j,i,i'} d_{i,i'}s_{i,j}s_{i',j+1} + 4(N-2)\lambda_A \sum_{i,j} s_{i,j} + \frac{w}{4}\sum_{j,i,i'} s_{i,j}d_{i,i'} + \frac{w}{4}\sum_{j,i',i} s_{i',j+1}d_{i,i'} - \theta\sum_j (s_{C,j}s_{D,j+1} + s_{D,j}s_{C,j+1}) \tag{13}$$

with $H = \sum_{iji'j'} J_{ij,i'j'}s_{ij}s_{i'j'} + \sum_{i,j} h_{ij}s_{ij}$, we have

$$\begin{cases} J_{ij,i'j'} = \delta_{i,i'} + \delta_{j,j'} + \frac{w}{4}((\frac{1}{2}(\delta_{j,j'-1}\, d_{i,i'} + \delta_{j,j'+1}\, d_{i,i'})) \\ -\frac{\theta}{2}((\delta_{i,C}\delta_{i',D} + \delta_{i,D}\delta_{i',C})\delta_{j,j'-1} + (\delta_{i,C}\delta_{i',D} + \delta_{i,D}\delta_{i',C})\delta_{j,j'+1}). \\ h_{ij} = 4(N-2)\lambda_A + \frac{w}{2}\sum_{i'} d_{i,i'} \end{cases} \tag{14}$$

Supplementary Note 7. Evolution of 81 spins in 9-city TSP

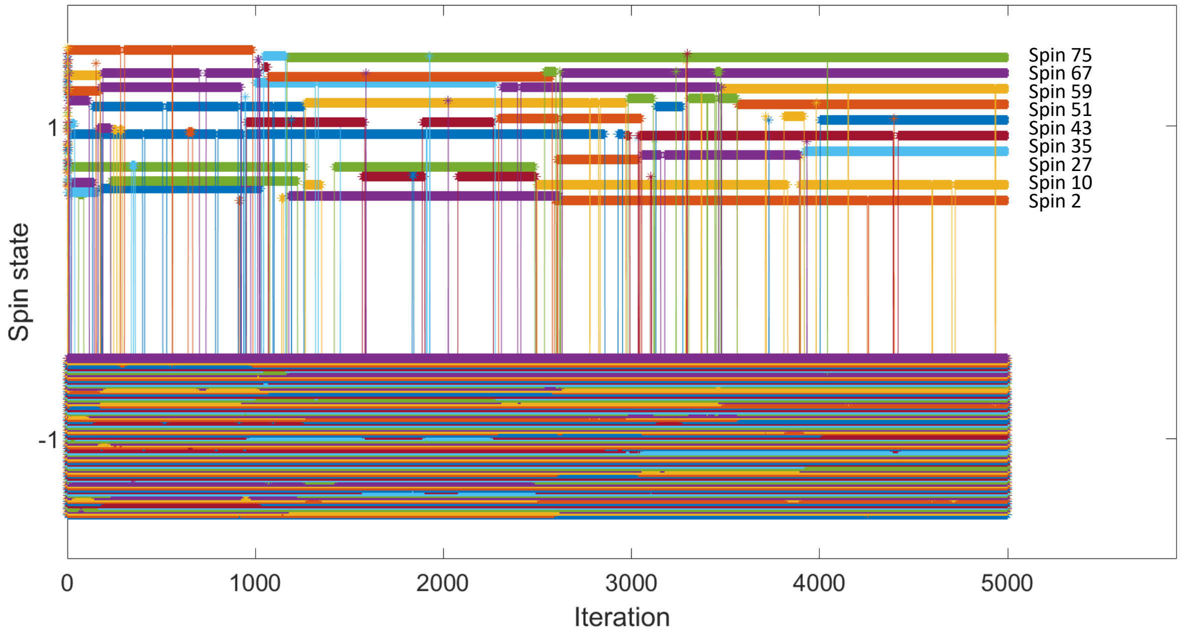

Supplementary Figure 7. Evolution of 81 spins in 9-city TSP problem. An offset is used in the *y*-axis to show states of each SMTJ clearly.

Supplementary Note 8. Discussion on inverse temperature *c* and parameter *w*

We run 10 different random initial states each with 5000 iterations. The effective inverse temperature *c* increases to 0.5 and hold. All cases can obtain near-optimal solutions, as shown in Supplementary Figure 8. However, there is a probability that the system jumps out of the ground state because of the none-zero temperature. If we continue to observe the evolution in a large timescale, the system would move back to the global minimum state.

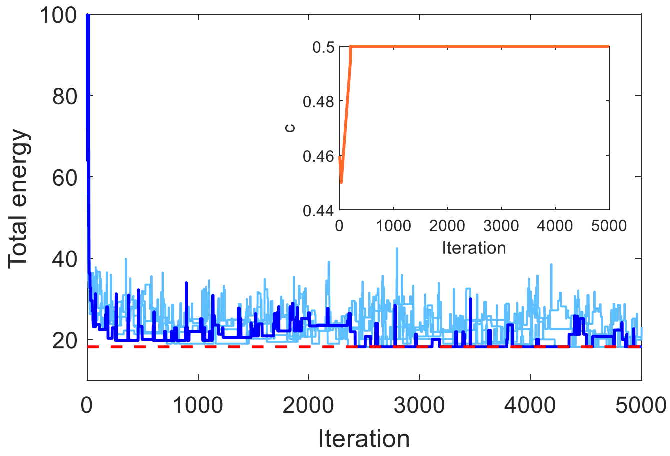

Supplementary Figure 8. Total energy transition of 10 solutions with randomly-generated initial states. Inset: effective inverse temperature (c) during computing. Red dashed line represents the ground state.

The parameter c which controls the global annealing should increase slowly. To evaluate the computation performance vs. time evolution of c, we test different time evolutions of c, as shown in Supplementary Figure 9. The initial value of c (c_initial) can be estimated as $\frac{1}{\Delta H} = \frac{1}{H_{max}-H_{min}} = \frac{1}{w \times N \times (\bar{d}-d_{min})} = 0.07 \approx 0.1$, where $w = 0.5$, $N = 9$ for a total of 9 cities, $\bar{d} = 4$ and $d_{min} = 0.8$ for the average and shortest distance of each two cities, respectively in Fig 3c. Note that this is a rough but universal estimation of the energy scale of c_initial. The actual optimal annealing temperature should depend on model details, and could be larger or smaller. In our experiments, we found that c_initial = 0.2 is more efficient through multiple trials, even though c_initial = 0.1 also converges to the ground state. For an Ising problem

whose answer is unknown, we can estimate the lower bond of c_initial by setting $w = 1$. It could be optimized further according to valid iterations and total time to solution. The final value of c (c_final) is set as the temperature under which all spins are “frozen”. The parameter c increases from the beginning to the end of computing. The total iterations should be much larger than the iteration of finding the optimal solution. The result shows that a rapid increase in c results in a poor probability of success.

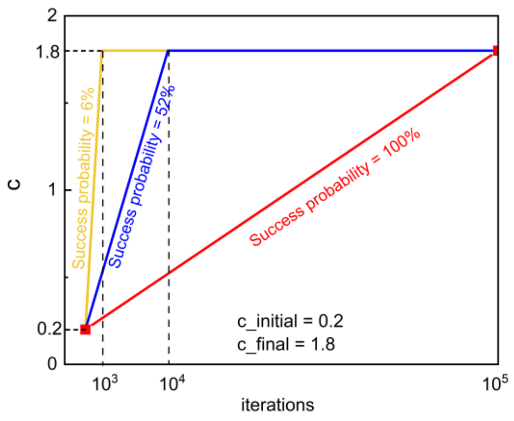

Supplementary Figure 9. Probability of success under different time evolutions of c.

The parameter w determines the relative strength of the constrain term and distance term. If the w is too large, then the probabilities of violations, namely the invalid path, would increase, as shown in Supplementary Figure 10. If the w is too small, then the effects of the distance term is small, which would lead to slower convergence to the ground state. The value of w can be chosen considering the percentage of valid iterations as well as the probability of success. We run the simulation of 9-city TSP for 30 times and find that the top value of probability of success exists around $w = 0.6$ within 50000 iterations. In most of our experiments, we choose $0.5 < w < 0.7$.

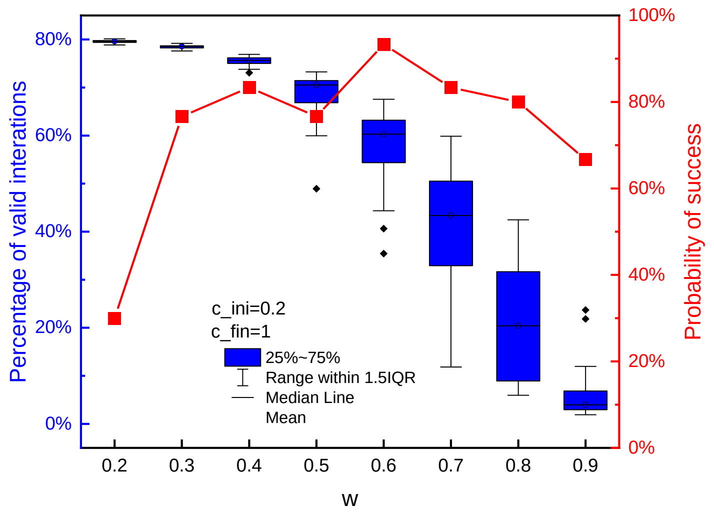

Supplementary Figure 10. Percentage of valid iterations and probability of success under different *w*.

Supplementary Note 9. Ising Hamiltonian of GP

We convert the clustering problem equivalent to the graph partitioning problem by creating an auxiliary graph $G_{GP}(V, E_{GP})$, where the binary edge is 1 if the corresponding edge in the original graph is below a threshold, otherwise, the edge is 0:

$$E_{GP,ij} = \begin{cases} 1, & if\ d_{ij} < d_{th} \\ 0, & otherwise \end{cases} \tag{15}$$

Typically, the threshold d_{th} depends on the diagonal length of the bounding box,

$$d_{\mathrm{th}} = \frac{\sqrt{(x_{\max} - x_{\min})^2 + (y_{\max} - y_{\min})^2}}{4} \tag{16}$$

The Hamiltonian for GP is $H_{GP} = A(\sum_i s_i)^2 + B \sum_{E_{GP,uv}=1} \frac{1-s_u s_v}{2}$. Here A and B are the weight constant for two terms, where the first term is the regularization term trying to ensure two sub-graphs with an equal size, and the second term is corresponding to minimizing the connections between two sub-graphs.

Particularly, in this 70-city TSP, partitioning into eight clusters is required since our Ising computer can handle a problem with no more than 80 nodes. As a result, we also need to partition the sub-graph until the size can meet the requirements.

Supplementary Note 10. Sliding window annealing based on CTSP

We integrate 8 neighbouring groups to a whole path by opening nearest two edges according to 8-city TSP sequence, as shown in Supplementary Figure 11. The initial path is obviously not an optimal path. We further optimize the whole path along its spatial dimensions (width and height) by carrying out constrained TSP over an input window. The window is shifted by 10 along each dimension in this TSP70 example and the size of the window is 40 × 40, as shown in Supplementary Figure 11. The path is cut to several sub paths by the window. Two longest paths are chosen and connected together as a circle path. Then we fix the visiting sequence of terminal points and ignore other sequences, and thus obtain a constrained TSP, as shown in Supplementary Figure 11a, where two green paths are fixed. After a new constrained TSP solving process, the path in the sliding windows is optimized as shown in Supplementary Figure 11b. Finally, we cut at green lines and reconnect to a whole path, as the blue lines and orange lines shown in Supplementary Figure 11b.

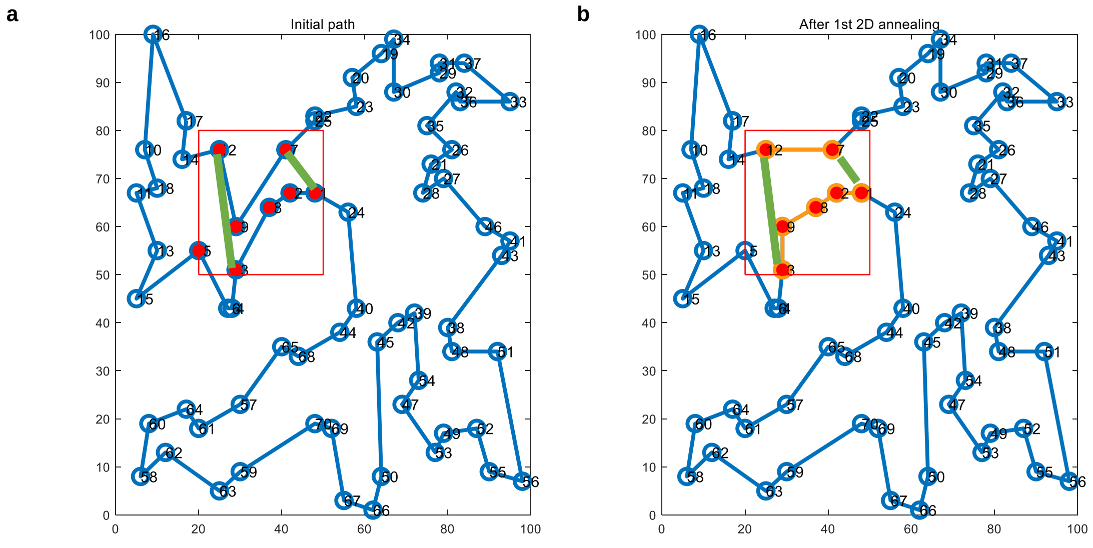

Supplementary Figure 11. Sliding window annealing and constrained TSP. a, 8 cities from the initial path chosen by a sliding window. **b**, optimized path within the sliding window after CTSP.

Note that we can change the size and step of the sliding window to achieve different accuracy at different annealing times. Supplementary Figure 12 shows the results of three times annealing. The result after the third time shows the near-optimal solution for 70-TSP.

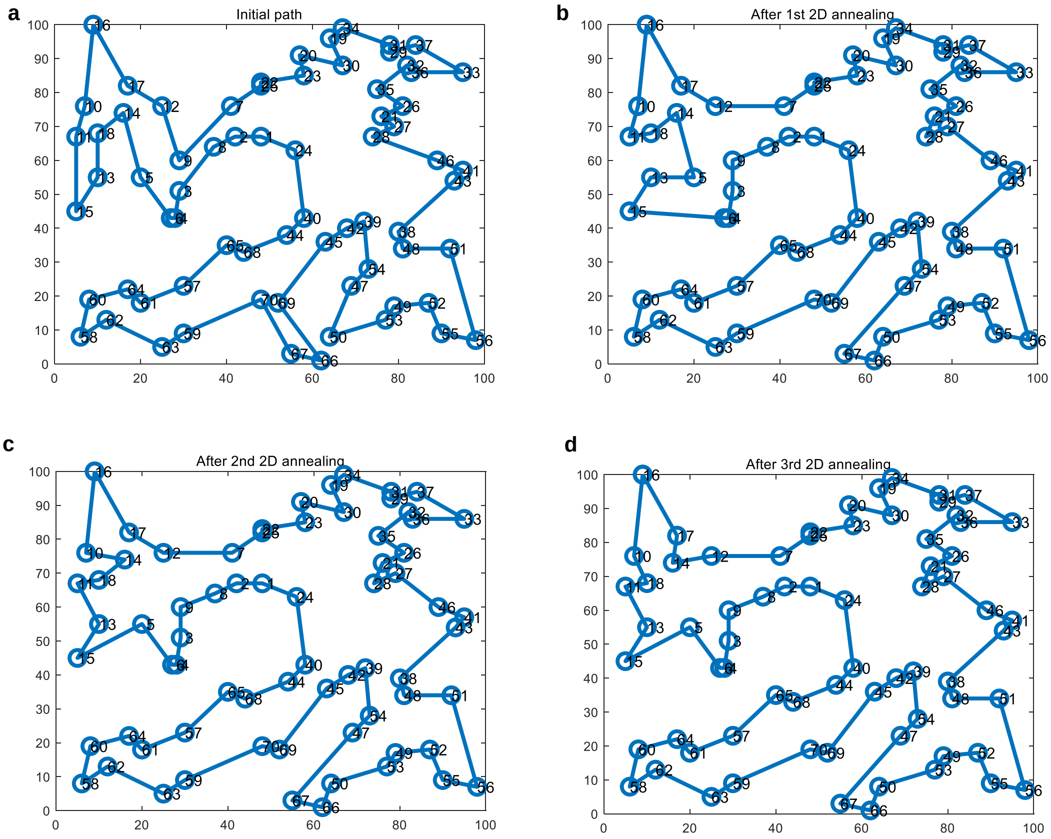

Supplementary Figure 12. Results of three times sliding window annealing. One annealing means optimization of the whole path for one time. Each annealing contains ~30 sliding windows (40 × 40, with a step of 10), and each window needs 4000~5000 iterations to solve CTSP. Thus, one annealing needs ~1.5×10^5 iterations. **a,** Initial path. **b,** Path after first annealing. **c,** Path after second annealing. **d,** Path after third annealing.

Supplementary Note 11. Quantitative analysis of scalability and implementation of 4 Kb SMTJ Ising computer

We discuss the scalability of our Ising computer by analysing performance metrics when solving N-city TSP with M-SMTJs in the array. The number of cities (N) and SMTJs (M) is summarized in Supplementary Table 1. The stochastic behavior of SMTJ is simulated according to Supplementary Eq. (17):

$$\text{SMTJ}=\mathit{sign}(2\times \mathit{rand}()-1+\tanh(I)) \tag{17}$$

where rand() is the function for generating random number from 0 to 1. I is the update current injected to SMTJ and sign() is the function for identifying the sign of the formula. The value of SMTJ (-1 or 1) represents its states (P or AP). The stochastic behavior of the SMTJ can be verified by randomly generating 100,000 states under different currents (I), according to Supplementary Eq. (17). The probability of state 1 (AP) is then counted and depicted in Supplementary Figure 13. The generated SMTJ states exhibit a probability distribution that closely aligns with the calibrated SMTJ behavior observed in our experimental results. Complete TSP task is performed in MATLAB, enabling the acquisition of performance metrics such as the solution quality, convergence speed, success rate and total number of iterations. All data during each iteration are recorded.

We construct a 4 Kb SMTJ Ising computer to discuss the hardware scalability, as shown in Supplementary Figure 14a. This system is divided into 4 PEs, each PE contains 1024 SMTJs organized as a crossbar array. These SMTJs function as stochastic Ising spins, mirroring their counterparts in experimental setups. The primary distinction lies in the design of the write/read circuits and control mechanisms, as these SMTJs are configured in a crossbar architecture. The stochastic behavior for circuit simulation is described in Verilog A according to Supplementary Eq. (19), with the resistance of 10 kΩ (P state) and 20 kΩ (AP state). The bit line (BL) is designed as 8 columns (3-bit BL) and 16 columns (4-bit BL) per PE for different scenarios. Each column is assigned a read sense amplifier (RSA). One row of SMTJs of each PE is read

per clock cycle. All peripheral circuits and NMOS can be implemented by Application-Specific Integrated Circuit (ASIC). Supplementary Figure 14b shows the 4-bit BL system, which has 64 columns (RSAs) in total, and needs 64 clock cycles to read all 4 Kb SMTJs.

Here is how we use this 4 Kb array when solving an Ising problem with a simple sequential mapping: First, the problem is mapped to an Ising model, and the parameters <J, h> are calculated and stored. Second, one row of SMTJs of each PE is selected and corresponding Vin are sent to SMTJs by DAC. SMTJs will fluctuate under Vin for $t > \tau 0$ ($\tau 0$ is the retention time), and their states are readout (Vout = Vdd or 0 V) through RSA. After reading one row of SMTJ, matrix multiply calculates the partial sum of Vin for the next iteration according to Supplementary Eq. (6), as shown in Supplementary Figure 14c. After the first row has been retrieved, the same process for the second row of each PE can be started, so and so forth. After M SMTJs are written and read, one iteration is finished, and all the partial sums have been added up to the next Vin. Then DAC will prepare the next row of Vin to the RSAs parallelly each clock cycle. After accessing all rows of the 4 Kb array, the system starts from the first row again. After multiple iterations, the states of SMTJs can be observed as the solution to an Ising problem.

Hardware simulations of the 4 Kb SMTJs Ising computer are carried out through Cadence Virtuoso software, using a commercial 40 nm 1P8M CMOS technology. The supply voltage of the system is 1.1 V. Simulation results verified the circuit design of the system. By carefully designing the size of transistors, a large range of linear current region of 1~15 µA flows through SMTJ is achieved, which exactly covers SMTJs in our experiments considering the variations, as shown in Supplementary Figure 14d. Transient simulations of the clock cycle of 0.1 ms are shown in Supplementary Figure 14e, which is limited by the retention time of SMTJ from our experiment (the longest retention time of SMTJ is 0.12 ms). The stochastic window in Supplementary Figure 14e demonstrates the stochastic transforming of an SMTJ from AP state

to P state. The main frequency of the SMTJ Ising computer could be further improved by using ultra-fast SMTJ with a smaller retention time, for example 8 ns, where the delay of read circuits would limit the system speed. Supplementary Figure 14f shows the delay of our current circuit design is around ~0.9 μs clock cycle, which could be further improved.

The energy consumption of the peripheral circuit including reading M SMTJ states, operating Matrix-Multiply, sending instructions to DAC, converting digital input to analogue signals and other control logic is obtained from hardware simulations. For matrix multiply operation, 64×64 16-bit multipliers are included to match our 4-bit BL system. Together with the average total number of iterations obtained from MATLAB simulations (equivalent of reading 4 Kb Ising computer for ~8000 iterations), the total time to solution and average energy consumption for solving a complete 70-city TSP task with a near-optimal solution of 695 can be estimated in Table 1 and Supplementary Table 2. The energy consumption of matrix multiplication is estimated as follows: 1) Estimate the power consumption of N-city TSP based on the power consumption of multiplication array. 2) Multiply the power consumption with the corresponding latency and iterations, then the energy consumption of each N-city TSP is derived. 3) Multiply the energy consumption of N-city TSP and the total number of corresponding N-city TSP required to solve the 70-city TSP. 4) Sum up the energy consumption calculated in 3) to obtain the total energy consumption. Here we provide two types of SMTJ with different retention times for simulations. One is SMTJ from our experiment (option 1, retention time of 0.1 ms) and the other one is ultra-fast SMTJ[6] (option 2, retention time of 8ns). The quality of the solution obtained from simulations using 4 Kb SMTJ Ising computer is better than the solution demonstrated in our experiment. The energy efficiency of option 1 is slightly higher than the experiment because of less power consumption of the cross-bar architecture. In addition, if we use ultra-fast SMTJ, the power consumption remains the same, while the total energy consumption of the main compute kernel and time to solution per

task would decrease to 1.84×10^{-4} J and 0.64 s, respectively. Meanwhile, the energy consumption of DAC would be greatly reduced because of less hold on time.

Each PE has 16×64 SMTJ array (Bit line BL [15:0], Word line WL [63:0]). If we implement this PE within 128 μm×11.2 μm, then the bit line/word line pitches are 1.6 μm/1 μm, respectively. From the Calibre parasitic extraction (TSMC40nm 1P8M CMOS), the coupling capacitance (Cc) of two adjacent BLs (64 μm long) is 4.80×10^{-16} F; at 1.1 MHz, its impedance |Zc| = 3.31×10^{8} Ω, which is high enough and capacitive cross talk is negligible. As the WL is even shorter (11.2 μm), its impedance capacitance is even higher and the capacitive coupling between WL is negligible as well. As for the inductive cross talk, the parasitic inductive of bit line L_C is 2.605×10^{-10} H and the mutual inductance between two adjacent BL cannot be extracted since it is even smaller than the threshold that the Caliber tool can extract; hence the mutual inductance is negligible as well. Additionally, its impedance (Z_L) is too low to cause any bouncing/mismatch at 1.1 MHz. The cross talk would be even lower at a main frequency of 10 kHz. As for the coupling through a resistance change, the structure of SMTJ array shares more similarity with that of NOR flash memory than that of NAND flash memory. The word lines (WLs) are selected sequentially, with only one WL being activated and read at a time. Consequently, the coupling through the resistance change between SMTJs within a single bit line (BL) is naturally avoided. Although there might be resistance coupling within a WL, each SMTJ channel is independently controlled by the respective RSA (read sense amplifier) through Vin [0:7]. Additionally, the EQ (Equivalent circuit) resets the initial voltage in the RSA circuit during PH0. Therefore, the impact caused by resistance coupling between SMTJs can be considered negligible.

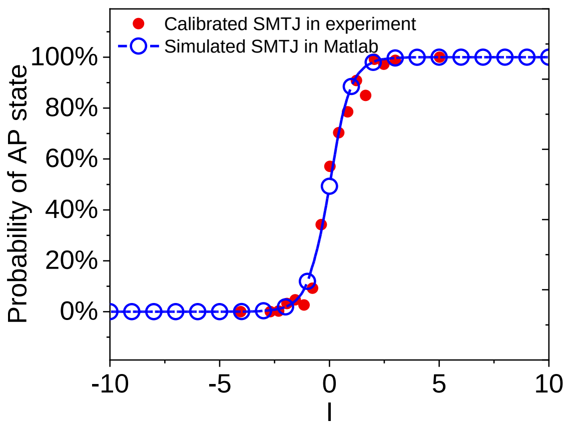

Supplementary Figure 13. Simulated stochastic behavior of SMTJ in MATLAB.

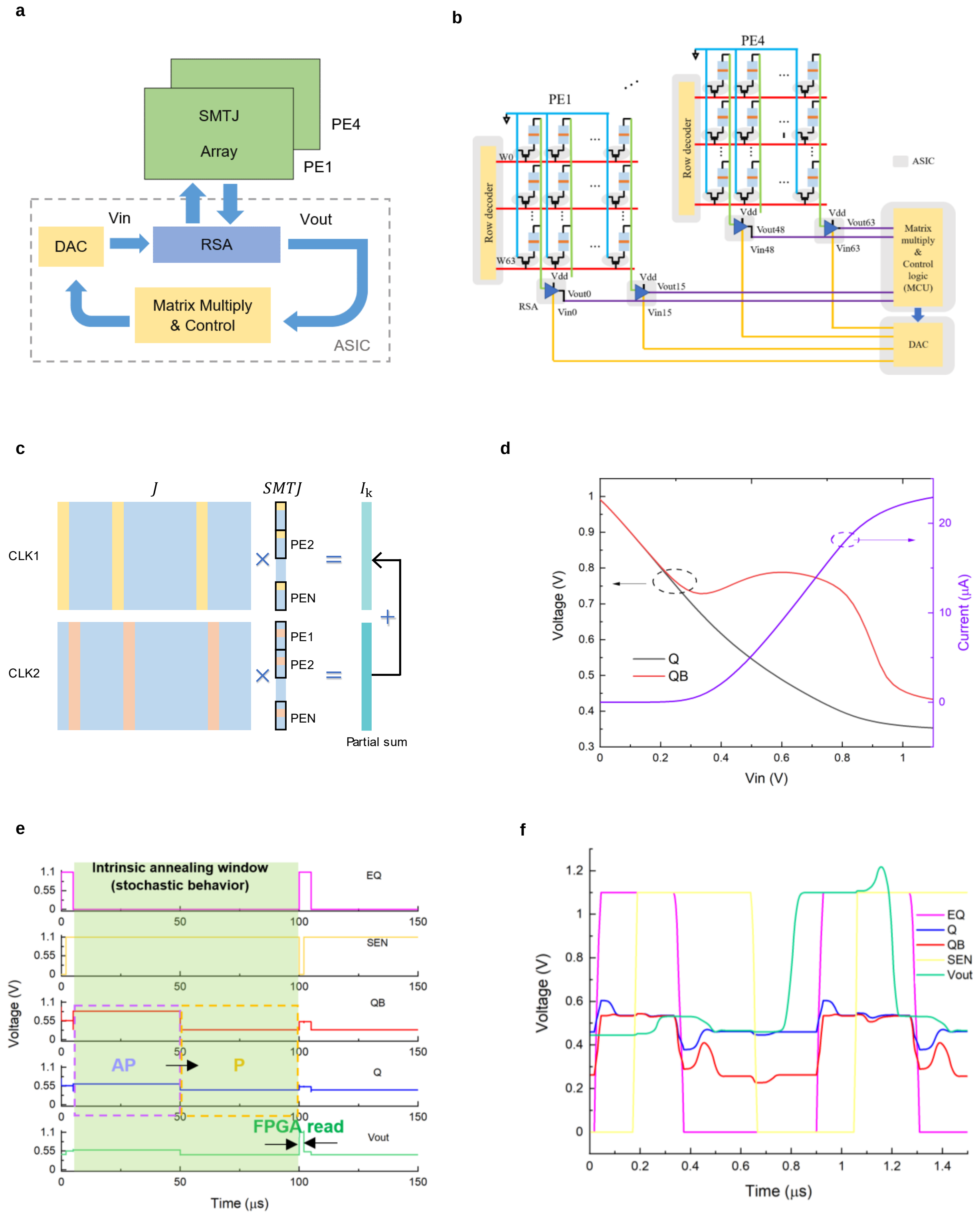

Supplementary Figure 14. Results of 4 Kb SMTJ Ising computer. a, Schematic of SMTJ system. **b**, 4 Kb SMTJ-based system with 128 rows and 8 columns (3-bit BL) per PE. **c**, Pipeline computing of update current (voltage). **d**, Simulated voltage of Q and QB point and current flow through SMTJ path when SMTJ is in the P state. **e**, Signals of the system frequency of 10 KHz. **f**, Signals of the system with a main frequency of 1.1 MHz.

Supplementary Table 1. Number of cities (N) of TSP and number of SMTJs (M) in the array in simulations

Dataset from TSPLIB	Number of cities (N)	Number of SMTJs (M) in the array		
St70	70	81	256	512
Eil101	101	81	256	512
KroA200	200	81	256	512

Supplementary Table 2. Energy consumption for solving 70-city TSP with a length of 695 using 4 Kb SMTJ Ising computer (simulations)

Technology			40 nm CMOS process	
Device			SMTJ with retention time of (0.1 ms in option 1; 8 ns in option 2)	
Cell type			1T1SMTJ	
Capacity			4 Kb, 4-bit BL	
Supply voltage (V)			1.1	
Write/Read current (μA)			1~14	
			Option 1	Option 2
Chip area (μm^2)			12,288	12,288
Energy to solution per task	Main compute kernel	SMTJ array (SMTJs and NMOS)	14.56 mJ	0.184 mJ
	Peripheral circuit	Row decoder	29.9 μJ	2.2 μJ
		RSA	0.045 J	0.00057 J
		DAC	0.322 J	0.004 J
		Matrix-multiply	96 μJ	
		Others (control and storage)	2.49 μJ	

Supplementary Note 12. Best solutions to TSP St70, Eil101 and KroA200

We show the best solutions we obtained when solving St70, Eil101 and KroA200 TSP. The best path from TSPLIB is 675, 642.3095 and 29368.

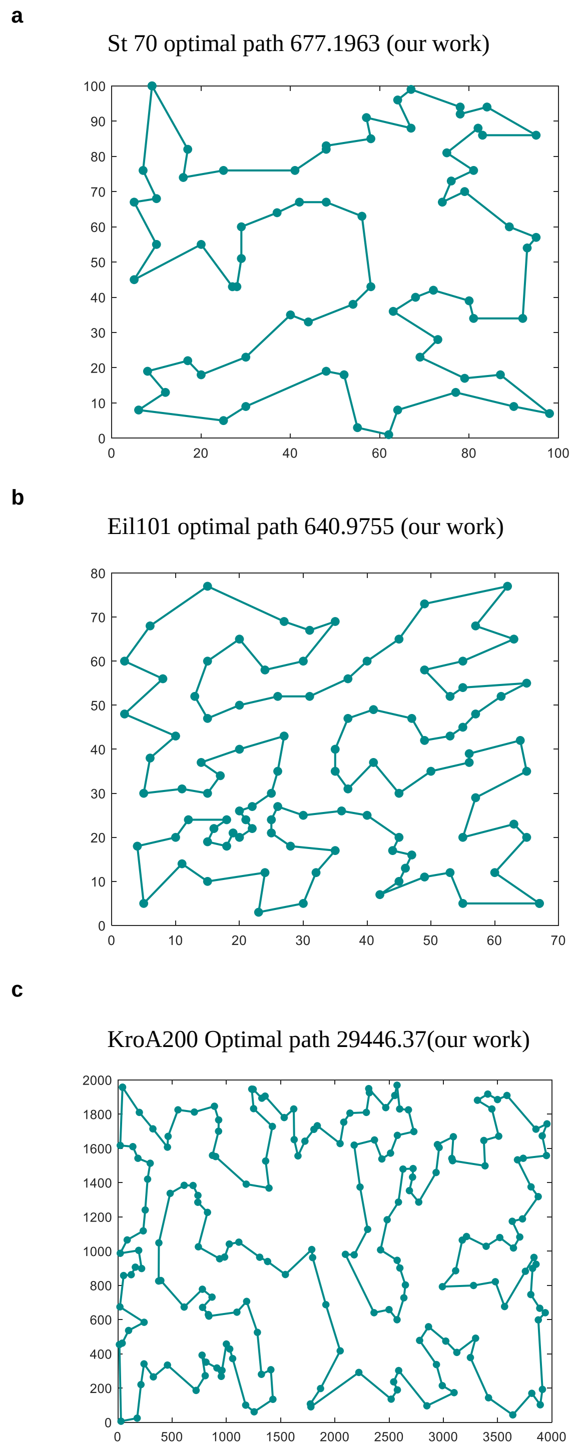

Supplementary Figure 15. Best solutions to TSP in our work. a, Optimal path for St70 problem. **b,** Optimal path for Eil101 problem, **c,** Optimal path for KroA200 problem.

Supplementary Note 13. Performance comparison with state-of-art Ising approaches

We compare the time to solution of state of art Ising approaches for solving 70-city TSP. We normalize the time to solution by node numbers and cycle numbers and then estimate 80-node Ising system to solve the complete TSP70 task with 40,000 cycles, simply assuming the time increases linearly with node and cycle numbers. These cycles include 2×10^4 iterations for dividing 70 cities into 8 groups using GP; 8×10^4 iterations for solving 9-city TSP within 8 groups; and 3×10^5 iterations for CTSP annealing.

For Quantum annealers[7,8], the time to solution of N = 55 MaxCut problem is 10^4 s, therefore the time to solution of TSP70 would be larger than 10^4s. For 2000-node CIM[9], the round-trip time is 5 μs in experiments, and thus the total solution time of TSP70 is estimated to be $5\ \mu s/2000 \times 80 \times 400000 = 80\ \mathrm{ms}$. Current CIM implementations are just proof of concept implementations and hence not yet optimized for energy efficiency. The mem-HNN approach runs at a main frequency of 1~10 MHz by using a 80-nm CMOS-memristor chip and estimates a main frequency of 500 MHz by using 16-nm CMOS technology[10]. Thus, the total solution time of TSP70 would be $1\ \mu s/100 \times 80 \times 400000 = 320\ ms$. In the phase-transition nano-oscillators approach, the delay is mainly due to the wire capacitance when the problem scale is large with all-to all connections. For an 8-spin system with 50%-connectivity (14-connection) in Ref.[11], the main frequency is $3.7\ \mathrm{ms}/250\ \mathrm{cycles} = 68\ \mathrm{KHz}$. Therefore, we assume a 80-spin PTNO system ($80 \times 79/2$=3160 connection) with a main frequency of $68\ \mathrm{KHz} \times \frac{14}{3160} = 301$ Hz, which would take 1.3×10^3 s (400000 cycles/301 Hz) to solve TSP70.

The total energy consumption per task of SMTJ-based approach for solving 70-city TSP in our experiment is calculated as $E = 80 \times 0.8\ \mathrm{V} \times 10\ \mu A \times 40 s = 25.6\ mJ,$ including transistors ($80 \times 0.4\ \mathrm{V} \times 10\ \mu A \times 40s = 12.8\ \mathrm{mJ}$) and resistors ($80 \times 0.25\ \mathrm{V} \times 10\ \mu A \times 40s = 8\ \mathrm{mJ}$). Energy consumption of other components is listed in Supplementary Table 3. We obtain the average power of DAC and MCU from the manuals.

Supplementary Table 3. The energy consumption for solving complete 70-city TSP task (experiment)

Part name	Energy consumption
SMTJ (containing transistor and resistor)	$80 \times 0.8\ \mathrm{V} \times 10\ \mathrm{uA} \times 40\ s = 25.6\ mJ$
DAC	$4 \times 80\ \mathrm{mW} \times 40\ s = 12.8\ J$
MCU	$8\ \mathrm{mW} \times 40\ s = 320\ mJ$
Others	$260\ mJ$
Total	$13.4\ J$

Supplementary References

1. Sutton, B., Camsari, K. Y., Behin-Aein, B. & Datta, S. Intrinsic optimization using stochastic nanomagnets. *Sci Rep* **7**, 44370 (2017).
2. Borders, W. A. *et al.* Integer factorization using stochastic magnetic tunnel junctions. *Nature* **573**, 390–393 (2019).
3. McMahon, P. L. *et al.* A fully programmable 100-spin coherent Ising machine with all-to-all connections. *Science* **354**, 614–617 (2016).
4. Zhou, A.-H. *et al.* Traveling-Salesman-Problem Algorithm Based on Simulated Annealing and Gene-Expression Programming. *Information* **10**, 7 (2018).
5. Bounds, D. G. New optimization methods from physics and biology. *Nature* **329**, 215–219 (1987).
6. Hayakawa, K. *et al.* Nanosecond Random Telegraph Noise in In-Plane Magnetic Tunnel Junctions. *Phys. Rev. Lett.* **126**, 117202 (2021).
7. Johnson, M. W. *et al.* Quantum annealing with manufactured spins. *Nature* **473**, 194–198 (2011).
8. Hamerly, R. *et al.* Experimental investigation of performance differences between Coherent Ising Machines and a quantum annealer. *Sci. Adv.* **5**, eaau0823 (2019).
9. Inagaki, T. *et al.* A coherent Ising machine for 2000-node optimization problems. *Science* **354**, 603–606 (2016).
10. Cai, F. *et al.* Power-efficient combinatorial optimization using intrinsic noise in memristor Hopfield neural networks. *Nat Electron* **3**, 409–418 (2020).
11. Dutta, S. *et al.* An Ising Hamiltonian solver based on coupled stochastic phase-transition nano-oscillators. *Nat Electron* **4**, 502–512 (2021).
